# A multi-ethnic genome-wide association study implicates collagen matrix integrity and cell differentiation pathways in keratoconus

**DOI:** 10.1038/s42003-021-01784-0

**Published:** 2021-03-01

**Authors:** Alison J. Hardcastle, Petra Liskova, Yelena Bykhovskaya, Bennet J. McComish, Alice E. Davidson, Chris F. Inglehearn, Xiaohui Li, Hélène Choquet, Mahmoud Habeeb, Sionne E. M. Lucas, Srujana Sahebjada, Nikolas Pontikos, Karla E. Rojas Lopez, Anthony P. Khawaja, Manir Ali, Lubica Dudakova, Pavlina Skalicka, Bart T. H. Van Dooren, Annette J. M. Geerards, Christoph W. Haudum, Valeria Lo Faro, Abi Tenen, Mark J. Simcoe, Karina Patasova, Darioush Yarrand, Jie Yin, Salina Siddiqui, Aine Rice, Layal Abi Farraj, Yii-Der Ida Chen, Jugnoo S. Rahi, Ronald M. Krauss, Elisabeth Theusch, Jac C. Charlesworth, Loretta Szczotka-Flynn, Carmel Toomes, Magda A. Meester-Smoor, Andrea J. Richardson, Paul A. Mitchell, Kent D. Taylor, Ronald B. Melles, Anthony J. Aldave, Richard A. Mills, Ke Cao, Elsie Chan, Mark D. Daniell, Jie Jin Wang, Jerome I. Rotter, Alex W. Hewitt, Stuart MacGregor, Caroline C. W. Klaver, Wishal D. Ramdas, Jamie E. Craig, Sudha K. Iyengar, David O’Brart, Eric Jorgenson, Paul N. Baird, Yaron S. Rabinowitz, Kathryn P. Burdon, Chris J. Hammond, Stephen J. Tuft, Pirro G. Hysi

**Affiliations:** 1 UCL Institute of Ophthalmology, London, UK; 2 Moorfields Eye Hospital, NHS Foundation Trust, London, UK; 3 Department of Paediatrics and Inherited Metabolic Disorders, First Faculty of Medicine, Charles University and General University Hospital in Prague, Prague, Czech Republic; 4 Department of Ophthalmology, First Faculty of Medicine, Charles University and General University Hospital in Prague, Prague, Czech Republic; 5 The Cornea Eye Institute, Beverly Hills, CA USA; 6 Department of Surgery and Board of Governors Regenerative Medicine Institute, Cedars-Sinai Medical Center, Los Angeles, CA USA; 7 Menzies Institute for Medical Research, University of Tasmania, Hobart, TAS Australia; 8 Division of Molecular Medicine, Leeds Institute of Medical Research, University of Leeds, Leeds, UK; 9 Institute for Translational Genomics and Population Sciences, The Lundquist Institute for Biomedical Innovation (formerly Los Angeles Biomedical Research Institute) at Harbor-UCLA Medical Center; Department of Pediatrics, Harbor-UCLA Medical Center, Torrance, CA USA; 10 Division of Research, Kaiser Permanente Northern California, Oakland, CA USA; 11 Department of Ophthalmology, Erasmus Medical Center GD, Rotterdam, The Netherlands; 12 Department of Epidemiology, Erasmus Medical Center GD, Rotterdam, The Netherlands; 13 Centre for Eye Research Australia, Royal Victorian Eye and Ear Hospital, East Melbourne, VIC Australia; 14 Department of Surgery, Ophthalmology, University of Melbourne, Royal Victorian Eye and Ear Hospital, East Melbourne, VIC Australia; 15 NIHR Biomedical Research Centre, Moorfields Eye Hospital, London, UK; 16 Amphia Hospital, Breda, The Netherlands; 17 The Rotterdam Eye Hospital, Rotterdam, The Netherlands; 18 Division of Endocrinology and Diabetology, Endocrinology Lab Platform, Department of Internal Medicine, Medical University of Graz, Graz, Austria; 19 Department of Ophthalmology, University Medical Center Groningen (UMCG), Groningen, the Netherlands; 20 Department of Ophthalmology, Academic Medical Center, Amsterdam, The Netherlands; 21 Vision Eye Institute, Melbourne, VIC Australia; 22 School of Primary and Allied Health Care, Monash University, Melbourne, VIC Australia; 23 Melbourne Stem Cell Centre, Melbourne, VIC 3800 Australia; 24 Section of Ophthalmology, School of Life Course Sciences, King’s College London, London, UK; 25 Department of Twin Research and Genetic Epidemiology, King’s College London, London, UK; 26 Department of Ophthalmology, St James’s University Hospital, Leeds, UK; 27 UCL Great Ormond Street Hospital Institute of Child Health, London, UK; 28 Oakland Research Institute, Oakland, CA USA; 29 Department of Ophthalmology, Case Western Reserve University, Cleveland, OH USA; 30 Centre for Vision Research, Department of Ophthalmology, Westmead Institute for Medical Research, University of Sydney, Westmead, NSW Australia; 31 The Jules Stein Institute, University of California Los Angeles, Los Angeles, CA USA; 32 Department of Ophthalmology, Flinders University, Adelaide, SA Australia; 33 Health Services and Systems Research, Duke-NUS Medical School, Singapore, Singapore; 34 QIMR Berghofer Medical Research Institute, Brisbane, QLD Australia; 35 St Thomas Hospital, Guy’s and St. Thomas NHS Trust, London, London, UK

**Keywords:** Corneal diseases, Genome-wide association studies

## Abstract

Keratoconus is characterised by reduced rigidity of the cornea with distortion and focal thinning that causes blurred vision, however, the pathogenetic mechanisms are unknown. It can lead to severe visual morbidity in children and young adults and is a common indication for corneal transplantation worldwide. Here we report the first large scale genome-wide association study of keratoconus including 4,669 cases and 116,547 controls. We have identified significant association with 36 genomic loci that, for the first time, implicate both dysregulation of corneal collagen matrix integrity and cell differentiation pathways as primary disease-causing mechanisms. The results also suggest pleiotropy, with some disease mechanisms shared with other corneal diseases, such as Fuchs endothelial corneal dystrophy. The common variants associated with keratoconus explain 12.5% of the genetic variance, which shows potential for the future development of a diagnostic test to detect susceptibility to disease.

## Abstract (web summary)

Alison Hardcastle et al. report a genome-wide meta-analysis of keratoconus, a condition affecting the cornea that causes blurred vision and often leads to blindness. They identify 36 genomic regions associated with keratoconus, 31 of which are novel, and show that the genes in these regions implicate genetic pathways involved in collagen matrix integrity and cell differentiation.

## Introduction

Keratoconus is a leading cause for visual impairment in adolescents and young adults which, untreated, can lead to legal blindness^1–7^. The prevalence of keratoconus varies between ethnic groups, with figures as high as 1.2% reported in some predominantly European populations^8^, to 2.3–3.3% in Maori or Iranian populations^9,10^. A high occurrence rate in first degree relatives, and concordance in twins, suggest that keratoconus has a strong genetic component^11,12^. Keratoconus can also be a comorbidity of other genetically determined conditions such as Down syndrome^13^. Several loci and variants for keratoconus have been identified through linkage studies and genome-wide association studies (GWASs) for central corneal thickness (CCT)^14–20^. However, although CCT is highly heritable, it is a stable characteristic, in contrast to the acquired and progressive corneal thinning that is a feature of keratoconus. Previous studies have also implicated single nucleotide polymorphism (SNP) alleles upstream of the *ZNF469* locus that is associated with a higher CCT but an increased risk for keratoconus^16,20,21^. Therefore, alternative mechanisms, in addition to those influencing CCT, are likely to be involved. This incomplete knowledge of the genetic predisposition for keratoconus limits our understanding of the mechanisms that drive this disease. In this study we present the largest GWAS for keratoconus performed to date for 4669 cases and 116,547 controls.

## Results

### Meta-analyses of genome-wide associations with keratoconus

**Fig. 1:**
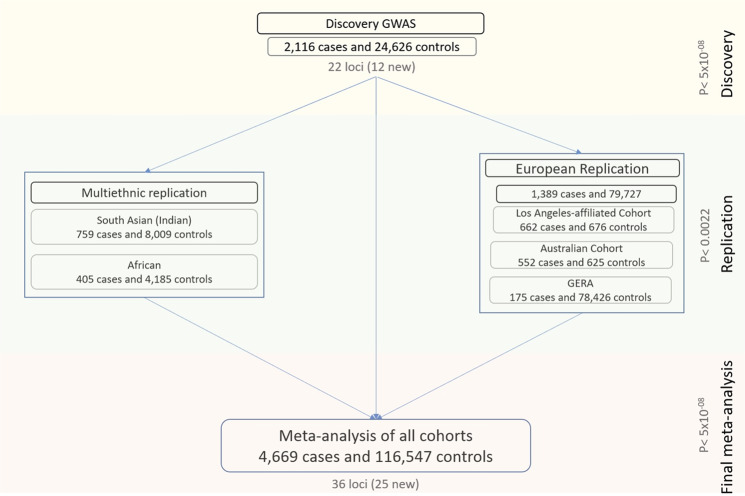
Work flowchart showing the flow of the genetic association analyses described in the manuscript. There were three main phases: a discovery in a European cohort of 2116 cases and 24,626 controls, a replication in a combined meta-analysis of three other European cohorts as well as in two smaller non-European cohorts, and a final meta-analysis involving all the multi-ethnic cases and controls from the previous stages.

We performed the analyses in three stages (Fig. 1). First, a discovery analysis was conducted in 2116 cases and 24,626 controls of European ancestry. For the second stage, we compared and replicated the discovery results in a meta-analysis of three independent European cohorts (1389 cases and 79,727 controls), and in a separate meta-analysis of two non-European cohorts (759 South Asian cases and 8009 controls, and 405 African cases and 4185 controls). Finally, we combined the discovery and replication cohorts in an overall meta-analysis. Genomic control factors^22^ were consistent with polygenicity expectations and the absence of uncontrolled population structure in any of the components of this study (Supplementary Data 1).

The discovery analysis identified 22 GWAS-significant associations (Supplementary Data 2), including six loci previously associated with keratoconus (*FOXO1*^17^, *COL5A1*^17^, *FNDC3B*^17^, *ZNF469*^17^*, LOX*^23^ and near *PNPLA2*^24^), four that were previously associated with CCT^16,18^ but not keratoconus, and 12 entirely novel loci. Among the novel keratoconus loci, the most significant association was found at a gene-poor region on chr21q2 (*p* = 1.34 × 10^−13^ for rs76747345).

**Fig. 2:**
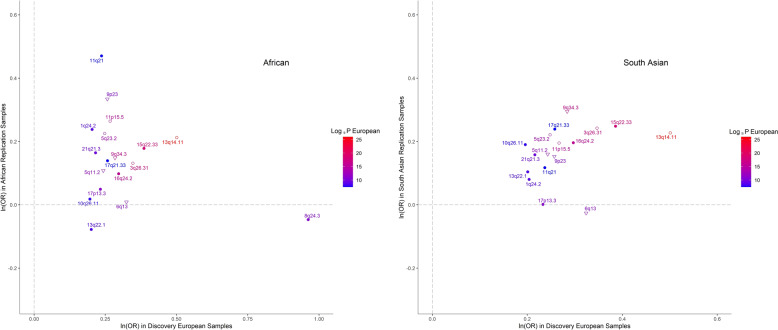
Comparison of effect sizes of associations for the same SNPs in individuals of African (405 cases and 4185 controls) and South Asian (759 cases and 8009 controls) ancestries. Each dot represents SNPs shown in Supplementary Data 3 and their labels are the respective chromosomal band on which they are located. The shapes of each data point refer to a previous GWAS association with keratoconus (empty circles), with CCT only (empty triangles) or novel associations (solidly filled circular shapes). The colors of both the points and their labels represent the significance (−log10(*p*-value) of association observed in the European discovery cohort. Polymorphisms identified in the discovery cohort but not shown here were not available for analysis in the replication cohorts.

At the replication stage, we carried forward the most significant SNPs within each of their regions of association, or other GWAS-significant proxy SNPs if necessary, whenever the index SNPs were missing in the replication data. Nine of the 22 regions (of which 2 are novel) replicated after Bonferroni multiple testing correction (*p* < 0.05/22 = 0.0022) and another four (of which 2 are novel) at FDR < 0.05 (Supplementary Data 2). All SNPs except three, for which the replication meta-analysis had insufficient power, showed directional consistency (Supplementary Fig. 1). We also observed associations that were highly directionally concordant in non-European samples (Fig. 2). Despite the lower statistical power, there was good replication in the South Asian samples (12 SNPs nominally significant, of which 8 remained significant after correction for multiple testing), but slightly less so in Africans (Supplementary Data 3).

**Fig. 3:**
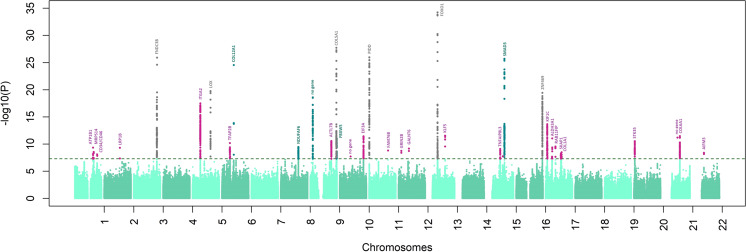
An annotated Manhattan plot of the final trans-ethnic meta-analysis data for all keratoconus cohorts in this study. Analyses were conducted in 4669 cases and 116,547 controls. The log10(*p*-value) from the final meta-analysis is shown on the y-axis for all the SNPs along the different autosomes (x-axis). Novel associations for keratoconus are in pink. The names of the coding genes nearest to the most significantly associated SNPs are shown, or “no gene” when the association was >250 kb from a coding gene. Different colors are used for genetic loci and genes that to our knowledge, were previously associated with CCT (dark green) keratoconus (dark gray).

The final meta-analysis combined data for all 4669 cases and 116,547 controls. Although the genomic inflation factor was nominally large (λ = 1.29), a further LD score regression analysis^25^ (on European samples only) suggests that these results were in line with expectations of polygenicity (ldsc intercept = 1.09, SE = 0.009). We continue to observe homogeneous effect sizes across all populations (Supplementary Data 4). This meta-analysis yielded significant associations clustering around 36 independent regions (Fig. 3, Table 1), of which 31 are reported for the first time, including six previously associated with CCT but not specifically with keratoconus at GWAS significance.

**Table 1:** GWAS the final trans-ethnic meta-analysis for keratoconus including all available cohorts, in 4669 cases and 116,547 controls.

Region	Cytoband	Chr.	Coordinates	SNP	A1	A2	log(OR)	log(OR) SE	*P*-value	Genes within the region
1*	1q24.2	1	169018902..169073504	rs1200108	A	G	−0.163	0.026	4.52E−10	***ATP1B1***
2*	1q25.1	1	174990905..175010262	rs6669560	T	C	0.156	0.026	2.92E−09	***MRPS14***
3*	1p22.2	1	207981419..208022291	rs761276	A	G	−0.137	0.024	8.02E−09	*CD34,****CD46***
4*	2q22.1	2	141867420..141878395	rs116792882	T	C	−0.686	0.110	4.82E−10	***LRP1B***
5	3q26.31	3	171565463..172000456	rs4894414	T	C	0.304	0.029	1.21E−26	***FNDC3B****,TMEM212*
6	5q11.2	5	52424518..52631507	rs12515400	T	C	0.217	0.025	3.16E−18	*FST,****ITGA2****,MOCS2*
7	5q23.2	5	121269498..121426675	rs840464	T	G	0.233	0.025	1.72E−20	*SRFBP1,****LOX***
8*	6p12.3	6	50788778..51623864	rs6904450	A	T	−0.189	0.029	6.44E−11	***TFAP2B****,PKHD1*
9	6q13	6	75746765..75834971	rs35523808	A	T	0.662	0.064	2.90E−25	***COL12A1***
10	8q22.1	8	95832448..95992020	rs1453379	T	C	0.156	0.025	3.32E−10	*TP53INP1,CCNE2,INTS8,****NDUFAF6***
11	9p23	9	13533300..13596674	rs1324175	T	C	−0.244	0.027	2.59E−19	No Gene
12	9q31.3	9	111372592..111500353	rs2417930	T	C	0.162	0.024	2.62E−11	***ACTL7B***
13	9q34.3	9	137412655..137566891	rs3118518	A	G	0.270	0.024	1.83E−28	***COL5A1****,RXRA*
14	9q34.3	9	139830733..139866247	rs11145948	A	G	0.173	0.025	9.89E−12	***FBXW5****,PTGDS,C8G,LCN12*
15*	10q21.1	10	55196113..55196113	rs117905623	T	C	−0.381	0.068	1.89E−08	No Gene
16*	10q26.11	10	120765171..120877372	rs658352	T	C	0.176	0.025	3.71E−12	*NANOS1,FAM45B,****EIF3A***
17	11p15.5	11	721570..825777	rs7117921	T	C	0.265	0.025	1.09E−26	*CEND1,TALDO1,EPS8L2,PDDC1,****PIDD***, *PNPLA2,RPLP2,SLC25A22*
18*	11q21	11	95308854..95308854	rs11021221	A	T	0.204	0.034	1.49E−09	***FAM76B***
19*	12p13.1	12	14288423..14290517	rs17340879	T	C	−0.493	0.082	1.77E−09	***GRIN2B***
20*	12q13.13	12	51754629..51756269	rs3782473	T	C	0.172	0.028	6.60E−10	***GALNT6***
21	13q14.11	13	41049191..41950539	rs2721051	T	C	0.452	0.037	5.71E-35	*MRPS31,MTRF1,NAA16,SLC25A15,ELF1*, ***FOXO1***, *WBP4*
22*	13q22.1	13	73634051..73649152	rs17285550	A	G	−0.181	0.026	2.84E−12	***KLF5***
23	15q21.2	15	51153797..51375011	rs11634895	A	G	−0.146	0.024	7.88E−10	*AP4E1,****TNFAIP8L3***
24*	15q22.2	15	61038143..61038143	rs76194223	T	C	0.258	0.046	1.75E−08	***RORA***
25	15q22.33	15	67438586..67694780	rs12912010	T	G	−0.312	0.029	1.99E−26	*AAGAB,IQCH,****SMAD3***
26	16q24.2	16	88274115..88344517	rs11117401	A	G	0.236	0.026	3.68E−20	*BANP,****ZNF469***
27*	17p13.2	17	4824625..4997433	rs12603055	C	G	−0.234	0.031	2.23E-14	*CHRNE,GP1BA,ZFP3,CAMTA2,ENO3,INCA1*, ***KIF1C***, *PFN1*,*RNF167*,*SLC52A1,SPAG7*
28*	17p11.2	17	19619441..19653310	rs4646785	T	C	−0.197	0.029	9.01E−12	***ALDH3A1****,SLC47A2*
29*	17q11.2	17	29883190..29889952	rs56161228	A	G	−0.210	0.033	2.70E−10	***RAB11FIP4***
30*	17q21.32	17	46328667..46645394	rs12948086	T	C	0.165	0.028	5.33E−09	*HOXB1,HOXB2,HOXB3,****SKAP1***
31*	17q21.33	17	48244531..48269903	rs2075556	C	G	−0.191	0.032	3.35E−09	***COL1A1****,SGCA*
32*	20p13	20	2076016..2143379	rs6106210	T	C	0.172	0.026	2.85E−11	***STK35***
33*	20q13.31	21	25357226..25357226	rs76747345	A	G	−0.545	0.080	6.53E−12	***NA***
34*	21q21.3	21	29521741..29623259	rs2143683	T	C	0.185	0.027	3.51E−12	No Gene
35*	21q22.3	21	47154348..47420667	rs142493024	A	G	0.760	0.111	9.07E−12	***COL6A1****,PCBP3*
36*	22q11.21	22	21322888..21323369	rs756878	T	C	0.159	0.027	4.01E−09	***AIFM3***

The field SNP lists the polymorphic variant showing the strongest association (P-value) for each region, for which the Chromosome number (Chr) and Coordinates (start and end coordinates in Human GHRC37/hg19) as well as the cytoband are shown. A1 lists the alleles at each SNP locus for which the effect sizes (as Odds Ratios, OR) and standard errors of estimates (log(OR) SE) are reported. Only coding genes nearest (<250 kb) to any significantly associated SNP are shown.

* Loci located within 1 million bp from loci previously associated at a GWAS level of significance (*p* < 5e−08) with keratoconus or CCT.

Bold characters indicate genes nearest to the SNP with the most significant association.

Strong associations were found near or within genes that code for fibrillar collagens (types I and V), microfibrillar (VI) and peri-fibrillar (XII) structures^26^, implicating impaired cohesion of the collagen matrix in the pathogenesis of keratoconus. Association was also found for rs35523808 (p.Glu2160Val, *p* = 2.90 × 10^−25^), a missense and potentially deleterious variant within *COL12A1* (Table 1, Supplementary Data 5). Collagen XII is localized in Bowman layer and the interfibrillar matrix of the corneal stroma, where it regulates the organization and mechanical properties of collagen fibrils^27^. We found significant association within the *COL6A1* gene (rs142493024, *p* = 9.07 × 10^−12^). The collagen VI protein is localized to corneal stroma filaments where it contributes to the integrity of the extracellular matrix^28^. Other associations were for *COL1A1* (rs2075556, *p* = 3.35 × 10^−09^) and *COL5A1* (rs3118518, *p* = 1.83 × 10^−28^). Collagen V is a regulator of collagen fibril formation, matrix assembly and tissue function in the corneal stroma^29^. Pathogenic variants in *COL12A1*, *COL6A1, COL1A1,* and *COL5A1* have been described in individuals with different subtypes of the connective tissue disorders Ehlers-Danlos Syndrome and osteogenesis imperfecta^30–33^.

We observed a strong association at the previously described^23^ *LOX* locus (rs840464 *p* = 1.72 × 10^−20^, Table 1). *LOX* encodes lysyl oxidase, an enzyme that initiates the cross-linking of collagens and elastin^34^. One of the associated SNPs at this locus, rs840462 (*p* = 2.08 × 10^−17^), displayed the most significant expression quantitative trait locus (eQTL) effects over the transcription of the *LOX* gene (*p* = 1.61 × 10^−13^) in a GTEx dataset for sun-exposed skin (Supplementary Data 6). A significant association was found near the integrin gene *ITGA2* (rs12515400, *p* = 3.16 × 10^−18^). The protein encoded by this gene participates in complexes of integrin α2β1, which are collagen receptors^35^ and which enhance type I collagen polymerization^36^.

We found association near the *ALDH3A1* gene (rs4646785, *p* = 9.01 × 10^−12^) for the same SNP that is also a significant eQTL, controlling transcription (eQTL *p* = 6.92 × 10^−10^) in sun-exposed skin (Supplementary Data 6) and other tissues. *ALDH3A1* encodes a corneal crystallin, a major component of the corneal stroma and epithelium that is upregulated in keratoconus corneas compared to controls^37^. It modulates corneal epithelial differentiation and homeostasis and may protect the eye from UV light-induced oxidative stress^38–42^.

For the first time, we report novel genetic associations implicating corneal cell differentiation and homeostasis in the pathogenesis of keratoconus. A significant association was found for rs17285550 (*p* = 2.84 × 10^−12^) near *KLF5*, a transcription factor that regulates corneal epithelial cell idenitity^43–46^. *KLF5* knockout mice have an abnormal collagen matrix^47^ and suppressed levels of *FNDC3B*, another gene associated with keratoconus in our study (rs4894414, *p* = 1.21 × 10^−26^). We found association for SNPs located near genes involved in fibroblast-keratocyte differentiation, such as rs761276 in the intergenic region between *CD34* and *CD46* (*p* = 8.02 × 10^−09^). CD34 is a negative marker of keratocyte differentiation in the cornea, expressed in stromal fibroblasts but not mature keratocytes^48^. Association was also found for SNPs located at the enhancer region of *SMAD3* (rs12912010, *p* = 1.99 × 10^−26^), a member of a family of genes involved in fibroblast differentiation^49^.

Association with several transcription factors involved in cell differentiation was revealed. The *NANOS1* and *EIF3A* genes are located in the same associated region (near rs658352, *p* = 3.71 × 10^−12^). *NANOS1* controls cell cycle progression and is involved in the *SMAD3*/TGFβ fibroblast maturation pathway^50,51^, while *EIF3A* is a cell differentiation suppressor^52^. *FOXO1* (rs2721051, *p* = 5.71 × 10^−35^) is implicated in the maintenance of keratinocyte stem cell identity^53^, and one associated region (rs12948086, *p* = 5.33 × 10^−09^) overlaps a cluster of *HOX* genes, that participate in early embryonic differentiation and morphogenesis^54^.

Our results show that two loci previously implicated in Fuchs endothelial corneal dystrophy (FECD)^55^ are also associated with keratoconus; the *PIDD1*/*SLC25A22* locus (rs7117921, *p* = 1.09 × 10^−26^) and *ATP1B1* (rs1200108, *p* = 4.52 × 10^−10^). The allele increasing the keratoconus risk at both loci also conferred susceptibility to FECD (Supplementary Data 7).

### Functional exploration of variants associated with keratoconus

Genes located in regions associated with keratoconus are broadly expressed across all GTEx^56^ and available eye tissues^57^ (Supplementary Fig. 2), more so in fetal corneas than in other eye tissues (Supplementary Data 8, Supplementary Fig. 3). In addition, certain genes near the association peaks were shown previously to be differentially expressed in keratoconus compared to control corneas^58^. Expression of *STK35*, that is associated in our analyses (rs6106210, *p* = 2.85 × 10^−11^), was increased two-fold in keratoconus corneas (FDR = 3.24 × 10^−03^) and *RAB11FIP4* (rs56161228, *p* = 2.70 × 10^−10^) found in our study, was increased by 2.4 fold (FDR = 3.24 × 10^−03^).

Gene-set enrichment analyses found associations with Gene Ontology annotations (Supplementary Data 9), including transcriptional regulation (*p* = 1.0 × 10^−06^), embryonic and primary germ layer development (*p* = 7.0 × 10^−06^), and embryonic morphogenesis (*p* = 3.0 × 10^−06^).

Because of limited availability of corneal eQTL datasets, we conducted a heritability-partitioning analysis to test for enrichment of genes in available eQTLs from other tissues^59^. The strongest enrichment, albeit not significant after multiple testing, were mainly collagen and fibroblast-rich tissues, such as aortic valve, myometrium, and skin (Supplementary Data 10).

We subsequently investigated the mechanisms through which the DNA variants associated with keratoconus in our meta-analyses alter the susceptibility to disease. We found that many of these variants contribute to epigenetic changes of the genomic regions in which they are located, alter the efficiency of transcription of nearby genes, or both (Supplementary Fig. 4). Mendelian randomization-based (SMR) tests^60^ which included all the SNPs for which we had summary statistics, regardless of the degree of genetic association with the trait, suggest that methylation is a widespread mechanism mediating the effect of disease-associated SNPs. We found evidence that SNPs associated with keratoconus often alter methylation of many genes, including *LOX, PDDC1, SMAD3, HOXB1, KLF5,* and *BANP* (Supplementary Data 11). Interestingly, methylation of *KLF4* was significantly (SMR *p* = 5.10 × 10^−08^) influenced by SNPs associated with keratoconus. *KLF4* is a transcription factor involved in tissue differentiation and development. It induces stem cell pluripotency in fibroblasts^61^ and regulates corneal epithelial cell cycle progression by suppressing canonical TGFβ signaling^62^ and is functionally related to *KLF5*, which specifies corneal epithelial cell identity^46^. We also found several eQTL mediated effects (Supplementary Data 12), which were less significant than the methylation-mediated effects.

### Inter-trait correlation and pleiotropy of keratoconus-associated loci

**Fig. 4:**
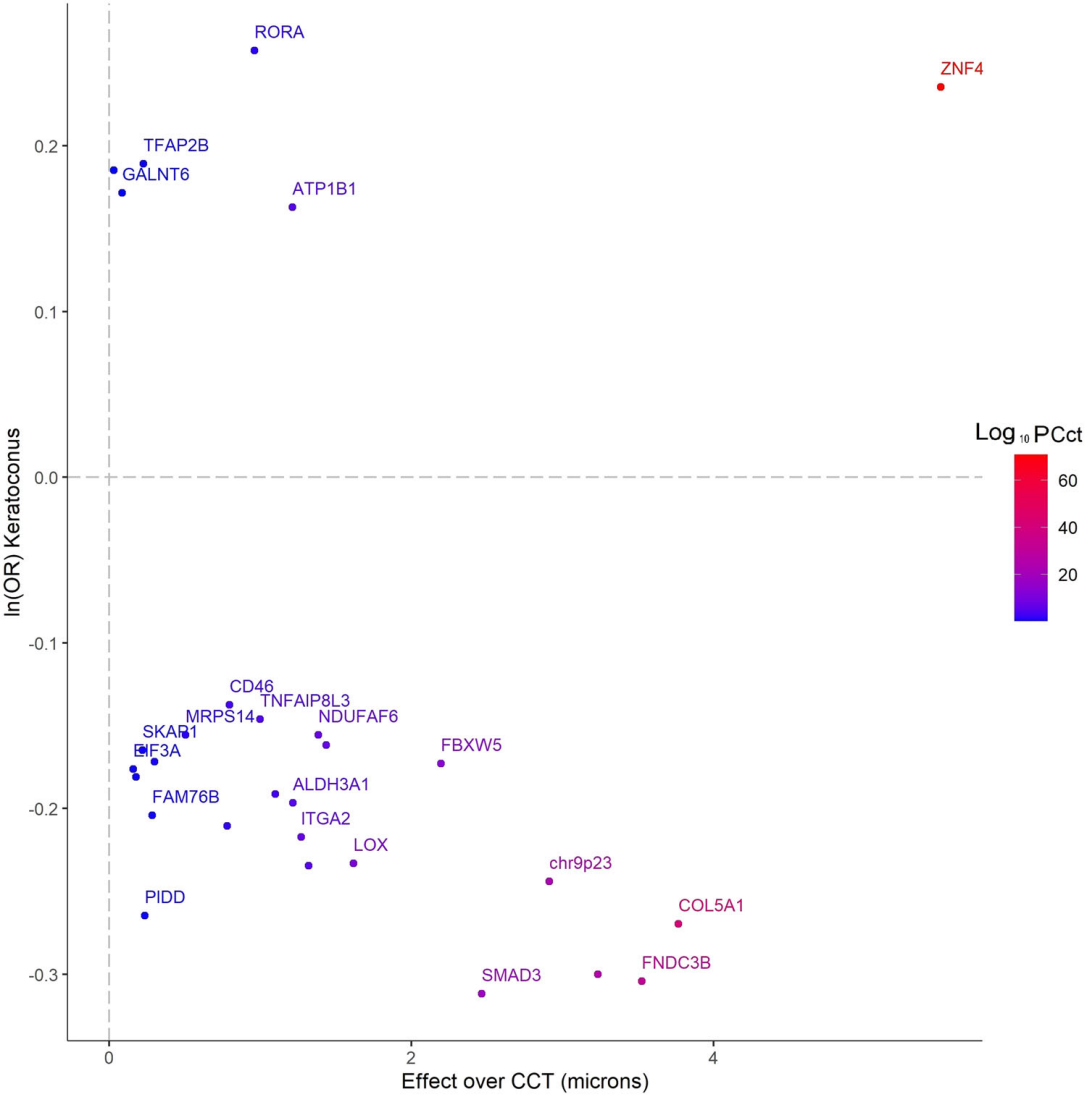
Comparison of the same SNP allele effect sizes over central corneal thickness (CCT; in microns, the x-axis). Data reported by Choquet et al.^20^ (also in Supplementary Data 14), with those over keratoconus (4669 cases and 116,547 controls, natural logarithm of the ORs, the *y*-axis). The alleles increasing susceptibility to keratoconus were selected as reference alleles. The genes located nearest to the most significant SNPs are shown in the labels and their color-codes denote the significance (−log10(*p*-value) of the association with CCT^20^. For the sake of clarity, only some of the genes are labeled. Unlabeled points denote SNPs in intragenic regions (distance from the nearest known protein-coding transcript greater than 250kbp). The variants are annotated to the nearest known protein-coding transcript. Because of their proximity with other SNPs, some loci in this figure are not labeled. Polymorphisms identified in the discovery cohort, but not shown in this figure were not available for analysis in the replication cohorts.

The strongest genetic correlations between keratoconus and other ocular traits (Supplementary Data 13) were with spherical equivalent (*p*_g_ = 2.07 × 10^−08^) and CCT (*p*_g_ = 2.5 × 10^−07^). However, as previously noted^16^, correlation of effect sizes with CCT was not always uniform. SNPs at the *ZNF469/BANP* locus, but also several loci that, to the best of our knowledge, are newly identified, such as the *ATP1B1* locus, diverged considerably from the linear correlation with CCT (Fig. 4, Supplementary Data 14). This supports the view that other mechanisms of loss of corneal integrity and corneal fragility, independent of corneal thickness, contribute to the pathogenesis of keratoconus. There were also nominally significant genetic correlations with asthma and ulcerative colitis (Supplementary Data 13), possibly because of the coincidental high collagen content in tissues involved in these diseases, or reflecting shared inflammatory components^63^.

### Predictive value of common genetic markers associated with keratoconus

**Fig. 5:**
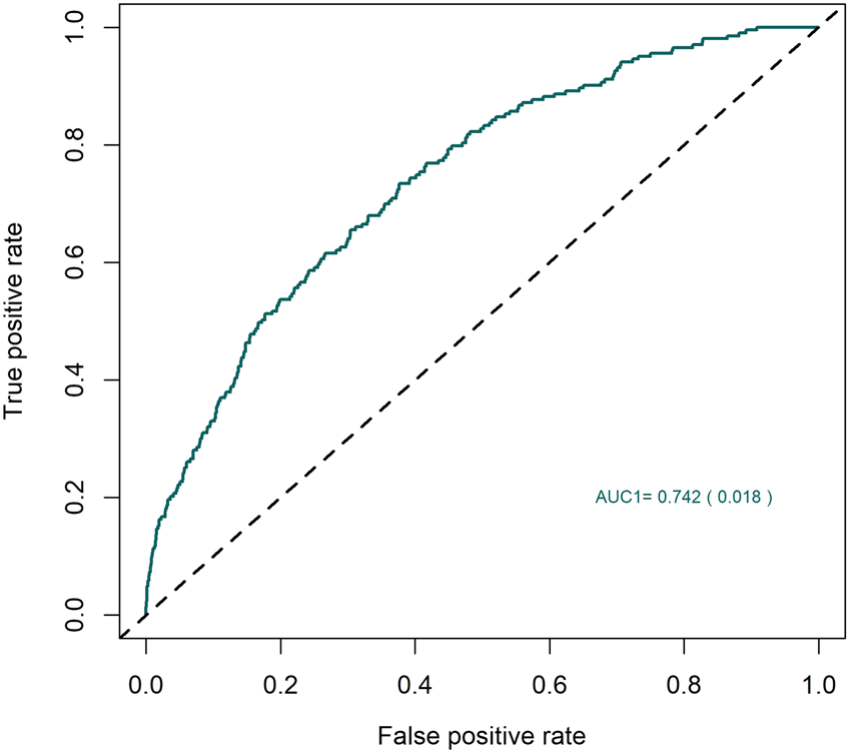
The predictive value of a genetic testing model based on common polymorphisms in an independent European case-control cohort. The area under the receiver operating characteristic curve for the performance of a keratoconus predictive model that used the SNPs identified through the multi-ethnic analysis. The model was trained in the discovery populations of European ancestry and was replicated in a completely independent European panel of 222 keratoconus patients and 2208 controls.

An LD score regression analysis revealed that genetic markers associated with keratoconus in our meta-analysis help explain 12.5% of overall keratoconus heritability among the same populations of European ancestry. Next, we assessed the predictive value of the markers we identified. A predictive model tested in a small, but independent panel of mixed British, Dutch and Austrian keratoconus patients and controls of European descent, found that the markers were moderately predictive (AUC = 0.737, SE = 0.017, Fig. 5) of keratoconus. The addition of rare variants, in future studies, is likely to further increase the predictive value of genetic testing.

## Discussion

In conclusion, we report 36 genetic loci strongly associated with keratoconus, 31 of which we identify for the first time. Their effect sizes are remarkably consistent across different ethnic groups (Supplementary Fig. 5). Larger studies are required to identify the remaining genetic risk for this condition (Supplementary Fig. 6). Our data highlight the importance of the integrity of the corneal collagen matrix in keratoconus. For the first time, we have identified a substantial role for cell differentiation pathways and stem cell regulators such as KLF4 and KLF5 in the pathogenesis of keratoconus, and a role for genes influencing connective tissue maturity.

Our study has several strengths. It is currently the largest of its kind which has allowed the identification of several loci, mostly novel, that predispose to keratoconus. In addition, although dominated by cases and control of European ancestry, the multi-ethnic component of our study allowed an evaluation of the strength of these associations among individuals of African and Asian descent. A limitation of this study is the lack of reliable corneal-specific eQTL and methylation level assessment. This has impeded the functional annotation of the discovered genetic loci, which are currently annotated to the nearest transcript-coding gene. Assuming that the effect of many SNPs is mediated through eQTL or methylation changes, cornea-specific analyses would improve these annotations. Lack of available tissue-specific expression and methylation data also currently limits our ability to further characterize these loci functionally. Our current approach would allow the identification of relationships that transcend tissue specificity and are observed in different cell lines. Also, the genomic inflation factor was moderately high, especially in our non-European cohorts, for which we are not able to fully evaluate the degree to which this is driven by polygenicity, a consequence of the presence of high-effect common variants in a low prevalence disease, or any other potential explanation.

Identification of genetic risk factors and novel disease mechanisms represents a substantial advance of our understanding of the corneal disease and, more broadly, connective tissue homeostasis, highlighting targets for the development of novel therapies. Two patient groups could directly benefit from an improved estimate of their risk of progressive corneal thinning. Firstly, individuals with subclinical keratoconus, in whom corneal collagen cross-linking would be an option to stop disease progression to reduce the need for contact lens wear for visual correction or eventual corneal transplantation. Secondly, genomic screening could be performed before laser refractive surgery for the correction of short sight (laser vision correction), to identify individuals at risk of severe visual loss from secondary progressive corneal thinning. Apart from refractive error, these individuals have normal eyes before surgery and there is currently no reliable mechanism to identify individuals at risk of developing this secondary corneal change, similar to keratoconus. For both groups, genomic data to estimate risk could be incorporated into models based on clinical parameters such as refractive error, corneal thickness, corneal shape, and corneal biomechanics^64,65^.

## Methods

### Study overview

This study was approved by the Institutional Review Board (IRB) or equivalent at all participating institutions, and participants provided written informed consent for the use of their genetic information. The study was conducted in concordance with the provisions of the Declaration of Helsinki.

Controls were extracted from a pool of 80,000 randomly selected participants in the UK Biobank cohort. Exclusions included any individual with any ICD9 or ICD10 code for any corneal disease. The cases and controls were ethnically matched.

### Multi-ethnic discovery cohort

#### Phenotyping

The majority of participants were recruited from specialist clinics at Moorfields Eye Hospital, London, UK. The study was approved by the Moorfields Eye Hospital Research Ethics Committee (09/H0721/19). The diagnosis of keratoconus was established based on clinical signs of corneal thinning and corneal distortion, with confirmation by corneal imaging (Orbscan, Bausch & Lomb, Rochester, USA, or Pentacam, Oculus, Wetzlar, Germany). Previous bilateral keratoplasties for keratoconus were also accepted as confirmation of disease status. Patients with keratoconus who had syndromic disease (e.g. Down syndrome, Leber congenital amaurosis, Ehlers Danlos syndrome) were excluded, as were patients with corneal dystrophy, of cornea guttata suggestive of coexisting Fuchs endothelial corneal dystrophy.

Recruitment from specialist corneal clinics at St. James’s University Hospital, Leeds, UK, was approved by Leeds East Research Ethics Committee (reference 10/H1306/63). The diagnosis of keratoconus was determined clinically and confirmed with corneal imaging (Orbscan, Bausch & Lomb, Rochester, USA, or Pentacam, Oculus, Wetzlar, Germany). Patients who had keratoconus associated with syndromes and those without the capacity to consent were excluded.

Participants were recruited from the Corneal and External Eye Disease clinics at Guy’s and St. Thomas’ National Health Service Foundation Trust, London, UK. The diagnosis of keratoconus was established based on a history of uni- or binocular progressive visual disturbance and the presence of some of all of a number of clinical signs seen on slit-lamp biomicroscopic examination including corneal stroma thinning, sub-epithelial iron deposition (Fleischer ring), stromal scarring, deep vertical stromal stress lines (Vogt’s striae) and corneal ectasia, with confirmation by three-dimensional corneal imaging (Pentacam, Oculus, Wetzlar, Germany). A history of previous bilateral keratoplasty for keratoconus was also accepted as confirmation of disease status. Patients with keratoconus who had syndromic disease (e.g. Down syndrome, Leber congenital amaurosis) were excluded.

Czech participants were selected based on the same criteria as used by Moorfields Eye Hospital London. The study protocol for Czech participants was approved by the Ethics Committee of the General Teaching Hospital and Charles University, Prague. Patients were recruited at the Department of Ophthalmology in Prague between 2011 and 2017. The diagnosis of keratoconus was based on the detection of localized steepening on corneal topography maps, together with localized corneal thinning in at least one eye. Only patients with KC grade 1 or higher according to the Pentacam (Oculus Optikgeräte GmbH, Wetzlar, Germany) build in software classification were included. Some eyes had advanced disease with typical signs such as Vogt striae, Fleischer ring, and stromal scarring, but this was not an inclusion requirement for the purposes of this study. Patients with bilateral keratoplasties for KC were also considered as cases. Patients with a known monogenic disorder were excluded.

The Melbourne patients were individuals with keratoconus and European background who were recruited from public clinics at the Royal Victorian Eye and Ear Hospital (RVEEH), private and optometry clinics in Melbourne, Australia. They were required to complete a study questionnaire, clinical examination, and details of family history of disease. A patient information sheet, consent form, privacy statement, and patient rights were provided to all individuals participating in the study. The study protocol was approved by the Royal Victorian Eye and Ear Hospital Human Research and Ethics Committee (Project#10/954H). Written informed consent was obtained from each participant and all protocols followed the tenets of the Declaration of Helsinki. A blood/saliva sample was also collected. DNA was extracted from the blood or saliva sample using NucleoSpin® QuickPure kits^66^. Keratoconus was diagnosed on the basis of the presence of one or more of the following: (1) an irregular corneal shape, as determined by distortion of keratometric mires and/or corneal tomographic images, (2) scissoring of the retinoscopic reflex; and (3) demonstration of at least one biomicroscopic sign, including Vogt’s striae, Fleischer’s ring or corneal thinning and scarring typical of Keratoconus^67^. Potential subjects with non-keratoconus ocular disease in both eyes such as keratectasia, corneal degenerations, macular disease, and optic nerve disease (e.g., optic neuritis, optic atrophy) were excluded from the study.

#### Genotyping

Both cases and controls were genotyped using the Affymetrix UK Biobank Axiom Array. For the discovery cohort cases, DNA was extracted from peripheral blood samples using standard methods (unless otherwise stated). DNA samples (*n* = 4,032) were quantified and normalized prior to genotyping using the Axiom 2.0 assay for GeneTitan on UK Biobank Axiom arrays (High-Throughput Genomics Centre at the Wellcome Trust Centre for Human Genetics, University of Oxford). 99% of samples reached the SNP QC call rate threshold of ≥97%. The procedures that produced the genotyping information in the UK Biobank participants that were used as controls for our analyses are described elsewhere^68^.

Intensity (CEL) files from cases and controls were called together using the “apt-probeset-genotype” program from Affymetrix (http://www.affymetrix.com/support/developer/powertools/changelog/apt-probeset-genotype.html, Thermo Fisher Scientific, Waltham, MA). Libraries were downloaded from the UK Biobank Axiom Array product support pages (https://www.thermofisher.com/order/catalog/product/902502). Controls genotyped using the UKBiLEVE SNP chip were removed from the calling process. Genotyping was done in batches of 4,000-5,000 samples each. We subsequently used SNP Polisher functionalities (probeset metrics, classification, OTV caller) following the Affymetrix recommendations (https://www.affymetrix.com/support/developer/powertools/changelog/VIGNETTE-snp-polisher-apt.html#otvcaller).

Post genotyping, UK Biobank sample genotypes were compared (via chi-square association testing) with the genotypes released from the UK Biobank. Only unrelated individuals (PI_HAT < 0.05) were included in the analyses. Subsequently, analyses were run in separate ethnic groups (African, South Asian, and Afro-Caribbean). SNPs with excessive (>0.05) missing genotypes and large (*p* < 0.01) differences in allele frequency compared with the official UK Biobank genotypes were removed from further analyses. This threshold of significance was adopted because even random missing samples that failed genotyping, either at the UK Biobank processing centre or in our labs, could cause minor and non-significant differences in allele frequencies.

#### Ancestry information and case-control matching

Cases and controls were subsequently matched for ancestry. First, a Principal Component Analysis (PCA) was run on independent (r2 < 0.3) directly genotyped SNP, then the samples were clustered in either one of the three major ancestral clusters: European, South Asian, or African. Samples that were not part of any of the clusters were removed from further consideration.

To avoid subtler population structure, arising either from ethnic heterogeneity or batch effects, we matched cases and controls based on the information from the first 10 principal components. We sought to match one case with up to 10 controls, if possible.

Only cases and controls that were matched in such a way were taken forward and imputed together.

#### Imputation

Imputation was run separately for the European and non-European ancestral groups (African and South Asian). The Europeans were split into two groups with an equal number of cases and controls and were imputed up to the HRC r1.1 2016 haplotypes using Minimac4 after Eagle 2.4 pre-phasing. Non-European ancestral groups were imputed up to the 1000 G Phase 3 v5.

#### Association analyses

Association analyses were conducted based on Firth’s regression models, given that our case-controls were imbalanced. Regression models were built with the keratoconus status as the dependent variable and the allele dosage at each SNP locus as a predictor. Analyses were adjusted for sex and the first 20 principal components and were conducted for each ancestral group separately. The largest case-control samples were of European ancestry and were used for discovery purposes. Samples of African and South Asian ancestries were used for validation purposes.

### Replication cohorts

#### The Los Angeles-affiliated cohort

##### Enrollment and phenotyping

Clinically affected keratoconus patients were enrolled into the study at three major sites: the longitudinal videokeratography and genetic study at the Cornea Genetic Eye Institute at Cedars-Sinai Medical Center, Los Angeles, CA, USA; the Jules Stein Eye Institute at UCLA, Los Angeles, CA, USA; and the University Hospitals Eye Institute at University Hospitals Case Medical Center and Case Western Reserve University, Cleveland, OH, USA. All patients diagnosed with keratoconus (see below) were offered recruitment into the study.

In addition to keratoconus patients, 126 local controls were recruited at Cedars-Sinai Medical Center. Convenience Caucasian controls from a Cholesterol and Atherosclerosis Pharmacogenetics (CAP) study were also included to make the sample size of controls equivalent to that of cases. CAP sample involved 944 unaffected volunteers, 609 of whom were self-reported white. Participants were aged 30 or above and were recruited from two clinical sites located in Los Angeles and San Francisco, CA, respectively. Additional details of CAP samples are described elsewhere^69^.

The diagnosis of keratoconus was performed by a corneal specialist ophthalmologist or an experienced research optometrist and based on clinical examination and videokeratography pattern analysis. Clinical examination included slit-lamp biomicroscopy, cycloplegic retinoscopy, and fundus evaluations. Slit-lamp biomicroscopy was used to identify stromal corneal thinning, Vogt’s striae, or a Fleischer ring. Retinoscopy examination was performed with a fully dilated pupil 20 min after phenylephrine 2.5% and cyclopentolate 1% drops had been instilled in the eye to determine the presence or absence of retro-illumination signs of keratoconus, such as the oil droplet sign and scissoring of the red reflex. Videokeratography evaluation was performed on each eye using the Topographic Modeling System (Computed Anatomy, New York, NY, USA), Orbscan II (Bausch & Lomb, Rochester, NY, USA), Oculus Pentacam (Oculus, Inc, Lynnwood, WA, USA) or Keratron (Optikon, Rome, Italy). Subjects were considered to have keratoconus if they had at least one clinical sign of keratoconus and a confirmatory videokeratography map with an asymmetric bowtie with a skewed radial axis above and below the horizontal meridian (AB/SRAX) pattern^70^. Importantly, topography was screened for mimicking disease such as pellucid marginal degeneration, which was excluded. Subjects that had bilateral keratoplasty for keratoconus were included if the surgical pathology report confirmed the presence of the disease.

##### Genotyping

DNA was extracted from EBV transformed lymphoblastoid cell lines established from peripheral whole blood of each study participant using NucleoSpin Tissue kit (MACHEREY-NAGEL Inc., Bethlehem, PA, USA) and from saliva samples using QIAsymphony DNA Kit (Qiagen, Germantown, MD).

Genotyping was performed using the Illumina HumanOmni2.5 Beadchip for all keratoconus patients, 126 local controls and 50 quality controls from CAP study. Genome-wide genotyping of half of CAP study subjects was performed using Illumina HumanHap300 BeadChip and of the remaining half of the samples with Illumina HumanCNV610-Quad Beadchip. Additional genotyping with iSelect Beadchip and Metabo-Chip was also available for some CAP samples. Samples with sex mismatches, relatedness (pi-hat>0.1875), low call rate (<95%), and SNPs with low minor allele frequency (MAF < 1%), low genotyping rate (<95%), and Hardy–Weinberg equilibrium p values less than 10^−6^ were excluded from the analysis. Both cases and controls datasets were imputed using the Michigan Imputation Server^71^ with the Haplotype Reference Consortium (HRC)^72^. Post-imputation QC removed SNPs with low imputation quality (rsq < 0.1) and a concordance rate less than 95% among 50 quality control samples. QC was performed using PLINK v1.9^73^. Principal components analysis (PCA) was performed with EIGENSTRAT^74^ Self-identified Hispanic samples were checked by PCA, and outliers significantly different from self-reported European individuals were excluded. After verifying the participants’ ancestry through a principal component analysis, 662 cases and 676 controls of full European ancestry were included in the analyses.

##### Association analyses

In total, 662 keratoconus cases and 676 controls (123 local controls and 553 controls from CAP study) with both phenotyping and genotyping data were available for analysis after QC. Under an additive genetic model, we conducted association tests between keratoconus and all autosome SNPs with MAF greater than 5%. The logistic regression model was performed adjusting for gender and three principal components (PCs) with RVTESTS^75^.

#### Australian cohort

##### Phenotyping

Genome-wide association analysis of this cohort has been described previously^24^. In brief, participants with keratoconus were ascertained through the eye clinic of Flinders Medical Centre, Adelaide; optometry and ophthalmology clinics in Adelaide and Melbourne; or an Australia-wide invitation to members of Keratoconus Australia, a community-based support group for patients. Approval was given by the Southern Adelaide Clinical Human Research Ethics Committee (HREC), the HREC of the Royal Victorian Eye and Ear Hospital and the Health and Medical HREC of the University of Tasmania. All participants gave informed consent and the study conformed to the tenets of the Declaration of Helsinki.

Patients were classified as having keratoconus if they had at least one clinical sign of keratoconus and a confirmatory videokeratography or a penetrating keratoplasty performed because of keratoconus, as described previously.^76^ The controls included 465 unaffected individuals from the Blue Mountains Eye Study^77^ and an additional 211 unaffected individuals^78^ from the Australian cohort previously described as controls in a GWAS for age-related macular degeneration (AMD) from the International AMD Genomics Consortium^79^.

DNA for cases and controls was extracted from whole blood using the QiaAMP DNA Maxi kit (Qiagen, Hilden, Germany).

##### Genotyping

Cases were genotyped for 551,839 variants using the HumanCoreExome array (HumanCoreExome-24v1-1_A, Illumina, San Diego, CA, USA) while for the controls, genotypes of 569,645 variants were generated with a customized Illumina HumanCoreExome array (“HumanCoreExome_Goncalo_15038949_A”) as described previously^79^. Only SNPs common to both arrays were included.

Quality control was carried out according to the protocol described by Anderson et al.^80^, modified as follows. Reverse and ambiguous strand SNPs were detected using *snpflip* (https://github.com/biocore-ntnu/snpflip, accessed March 24, 2017) and flipped or excluded. Ancestry outliers identified by principal component analysis (PCA) using EIGENSTRAT^74^, as well as individuals missing genotype rate > 0.05, heterozygosity more than three standard deviations from the mean, or discordant sex information, were excluded. Related individuals were detected by calculating pairwise identity by descent (IBD), and the individual with the lower genotyping rate in any pair with IBD > 0.185 was removed. Markers were excluded if they had significantly different missing data rates between cases and controls, total missing genotype rate > 3%, minor allele frequency (MAF) < 0.01, or deviated significantly (*P* < 10^−5^) from Hardy–Weinberg equilibrium. Following all exclusions, there were 522 cases (mean age 45) and 655 controls (mean age 65) genotyped for 264,115 common platform SNPs.

##### Association analyses

Genotypes of autosomal SNPs were phased with Eagle (version 2.3.5)^81^ and imputed to the EUR subset of the 1000 Genomes Project reference panel (Phase III, version 5)^82^ using Minimac3 (version 2.0.1)^71^. Indels, SNPs within 5 bp of an indel, rare variants (MAF < 0.01), and variants with poor imputation quality (*R*^2^ < 0.8) were excluded. A total of 6,252,612 including 250,964 genotyped variants, passed quality control. Association analysis was performed on most-likely genotypes under a logistic regression model using PLINK (version 1.90)^83^ using the first three principal components as covariates.

#### Genetic epidemiology research in adult health and aging cohort (GERA)

The Genetic Epidemiology Research in Adult Health and Aging (GERA) cohort is part of the Kaiser Permanente Research Program on Genes, Environment, and Health (RPGEH) and has been previously described in detail^84,85^. The GERA cohort comprises 110,266 adult men and women who are consented participants in the RPGEH, an unselected cohort of adult participants who are members of Kaiser Permanente Northern California (KPNC), an integrated health care delivery system, with ongoing longitudinal records from vision examinations. For this analysis, 78,583 adults, who self-reported as non-Hispanic white, were included. Of which 72 cases were males and 85 females, with an average age of 61.9 years (S.D. 12.3). All study procedures were approved by the Institutional Review Board of the Kaiser Foundation Research Institute.

##### Phenotyping

Keratoconus cases were identified in the KPNC electronic health record system based on the following International Classification of Diseases, Ninth Revision (ICD-9) diagnosis codes: 371.60, 371.61, and 371.62. All selected keratoconus cases (*N* = 157) had at least one diagnosis of keratoconus made by a Kaiser Permanente ophthalmologist. Our keratoconus control group (*N* = 78,426) included all the non-cases.

##### Genotyping

DNA samples from GERA individuals were extracted from Oragene kits (DNA Genotek Inc., Ottawa, ON, Canada) at KPNC and genotyped at the Genomics Core Facility of the University of California, San Francisco (UCSF). DNA samples were genotyped at over 665,000 single nucleotide polymorphisms (SNPs) on Affymetrix Axiom arrays (Affymetrix, Santa Clara, CA, USA)^86,87^. SNPs with initial genotyping call rate ≥97%, allele frequency difference ≤0.15 between males and females for autosomal markers, and genotype concordance rate >0.75 across duplicate samples were included^85^. Around 94% of samples and more than 98% of genetic markers assayed passed quality control (QC) procedures. In addition to those QC criteria, SNPs with genotype call rates <90% were removed, as well as SNPs with a minor allele frequency (MAF) < 1%.

Following genotyping QC, we conducted statistical imputation of additional genetic variants. Following the pre-phasing of genotypes with Shape-IT v2.r72719^88^, variants were imputed from the cosmopolitan 1000 Genomes Project reference panel (phase I integrated release; http://1000genomes.org) using IMPUTE2 v2.3.0^89–91^. As a QC metric, we used the info *r*^2^ from IMPUTE2, which is an estimate of the correlation of the imputed genotype to the true genotype^92^. Variants with an imputation *r*^2^ < 0.3 were excluded, and we restricted to SNPs that had a minor allele count (MAC) ≥ 20.

##### Association analyses

We ran a logistic regression of keratoconus and each SNP using PLINK^83^ v1.9 (www.cog-genomics.org/plink/1.9/) with the following covariates: age, sex, and genetic ancestry principal components (PCs). We modeled data from each genetic marker using additive dosages to account for the uncertainty of imputation^93^. Eigenstrat^74^ v4.2 was used to calculate the PCs^84^. The top 10 ancestry PCs were included as covariates, as well as the percentage of Ashkenazi ancestry to adjust for genetic ancestry, as described previously^84^.

#### UK samples of non-European ancestry

Among the subjects recruited at the Moorfield Eye Hospital, there were 759 cases of South Asian ancestry and 405 of African descent. Phenotypes were obtained as described before. These samples were matched from a separate pool of ethnic minorities from the UK Biobank and analyzed using Firth’s regression models, adjusting for sex and the first 20 principal components that were calculated within the ethnically matched case-control group. These samples were primarily used for replication and validation purposes, but also contributed to the final meta-analysis.

### Cohorts used for prediction

Prediction models were trained in cases and controls of European descent described above and tested in 222 keratoconus patients and 2208 keratoconus-free controls, pooled from the Erasmus Medical Centre cohort and the UK Biobank.

#### The Erasmus Medical Centre (EMC) cohort

The Erasmus Medical Centre (EMC) cohort consisted of Caucasian keratoconus patients (*n* = 156) and controls (*n* = 1476). Keratoconus samples were collected from both the Erasmus MC and the Rotterdam Eye Hospital. Control samples were collected from 1) the Rotterdam study (*n* = 448), a population-based study described previously^94^; 2) the Biomarkers of personalized Medicine (BioPersMed) study (*n* = 891) which included healthy subjects with one or more risk factors for cardiovascular diseases (Graz, Austria); 3) controls from the Amsterdam Glaucoma Study (*n* = 137), previously described^95^. All patients and controls underwent a full ophthalmic examination at the correspondent medical centers as part of the inclusion protocol of each study. The studies were approved by the Institutional Review Board at each institute and informed consents were collected from all participants.

DNA from cases and controls was genotyped using the Illumina bead chip (Infinium Global Screening Array-24 V2; Illumina Inc) at the Human Genetics facility at EMC. Quality control was performed using Genome studio (Illumina-designed software) and PLINK on 683880 genotyped variants from 1632 individuals. In summary, samples with more than 10% missing variants and variants with minor allele frequency less than 5% were excluded. Moreover, a Hardy–Weinberg equilibrium cut-off of 10^−7^ was used for control samples. The genomic inflation factor (lambda) of 1.08 (based on median chi-square), showing no significant dispersion of test statistics from the expected distribution. A total of 454925 variants (genotyping rate of 0.998) from 1629 subjects (155 cases and 1474 controls) were imputed using Michigan imputation server. Post-imputation quality checks were performed and a total of 57 variants from 1629 subjects were included in the final analysis.

#### The UK Biobank subset

A subset of 66 cases and 733 controls were extracted from the UK Biobank cohort. In brief cases were individuals who reported keratoconus (ICD code H18.6) and controls were UK Biobank participants who did not report keratoconus but also were negative for other significant corneal disease (ICD10 codes H18.7, H18.8 and H18.9) and without any prior history of eye surgery. UK Biobank participants that were included as controls in the previously described analyses were specifically excluded from this step. All methods, genotyping, imputation, and basic QC were similar to what is described elsewhere before^96^.

### Meta-analyses

We conducted three meta-analyses. For the initial meta-analysis (discovery), we used summary statistic results from the discovery cohorts. These cohorts were genotyped on the same chip and recruited using consistent methodologies. The second meta-analysis aggregated data from summary statistics of three independent cohorts of European ancestry, recruited in addition to the discovery cohort. For the final meta-analysis, we used all available information from all available cohorts. Very rare variants (MAF < 0.01) and rare ones (0.01 < MAF < 0.05) with low imputation quality scores (<0.8) or high meta-analysis heterogeneity *I*^2^ > 0.75 were excluded.

For all meta-analyses we applied a fixed-effect inverse variance method as implemented in the software METAL^97^ and GWAMA^98^. No genomic control adjustment was applied during the meta-analysis.

### Effective population size and power calculations

Power calculations were conducted using the Stata 15 “*power”*’ package (StataCorp LLC, College Station, TX).

The effective population size was calculated per each locus, aggregating the effective population sizes of each cohort participating in the meta-analyses, using this equation:

$${\mathrm{N}}.{\mathrm{eff}} = 4/\left( {\frac{1}{{{\mathrm{N}}.{\mathrm{cases}}}} + \frac{1}{{{\mathrm{N}}.{\mathrm{controls}}}}} \right)$$

as recommended elsewhere^97^, where N.eff is the effective sample size, N.cases is the number of cases, and N.controls is the number of subjects without keratoconus.

Only SNPs with minor allele frequency of at least 1%, which were available from at least 70% of the maximum number of participants across all studies, and that were not missing in more than one stratum (cohorts), were considered.

### Multiple testing correction

Two methods of correcting for multiple testing were used. The first was a classic Bonferroni correction, in which the threshold of significance (0.05) was divided by the number of tests (*n*):

$$\alpha = \frac{{0.05}}{{{n}}}$$

Given the large number of loci for which replication was needed, we additionally calculated the False Discovery Rates, using the Benjamini–Hochberg method^99^.

### Genomic inflation

To assess the potential inflation of association probabilities, genomic inflation factors^22^ were calculated and Q-Q plots were drawn using the package “gap” in R (https://cran.r-project.org/).

### LDscore regression-based methods

#### Calculation of genomic inflation attributable to polygenicity vs. stratification

We used LD score regression-based methods to calculate the genomic inflation factors, and measures of stratification within the samples of European ancestry. We followed the LDSC authors’ recommendations (https://groups.google.com/g/ldsc_users/c/yJT-_qSh_44/m/MmKKJYsBAwAJ) to normalize the effective sample sizes and account for the different numbers of cases and controls in the different populations, thereafter using a sample prevalence estimate of 0.5, and a disease prevalence in the population of 0.001.

#### Calculation of genetic correlation

Bivariate genetic correlations between keratoconus and other complex traits whose summary statistics are publicly available were assessed following previously described methodologies^100^, using the program LD Score (https://github.com/bulik/ldsc).

### Genesis

We used a maximum-likelihood model to estimate the distribution of effect sizes, based on summary statistics of observations and linkage disequilibrium patterns to predict the likely number of SNPs that explain keratoconus heritability as well as explore the relationship between future sample sizes and the number of SNPs identified and variance or heritability explained as described elsewhere^101^ and implemented in the GENESIS R package (https://github.com/yandorazhang/GENESIS).

### SNPs and gene annotations

Polymorphisms associated at a GWAS level (*P* < 5 × 10^−08^) were clustered within an “associated genomic region”, defined as a contiguous genomic region where GWAS-significant markers were within 1 million base pairs from each other, as suggested elsewhere^102^. Significant polymorphisms were annotated with the gene inside whose transcript-coding region they are located, or alternatively, if located between two genes, with the gene nearest to it. The associated genomic regions were collectively annotated with the gene overlapping, or nearest the most significantly associated variant within that region. In addition, the polymorphic sites were functionally annotated using SNPnexus^103^. CADD scores were generated using Ensembl variant effect predictor (http://grch37.ensembl.org/Homo_sapiens/Tools/VEP).

#### Previous association with keratoconus and CCT

We collected evidence of previous associations with keratoconus and CCT by querying the GWAS catalog^104^ and looking up reports in peer-reviewed articles^16,17,20,24,105^ for genomic markers and regions associated with either. We specifically looked for genome-wide significance level of association.

### SMR

SMR (Summary data-based Mendelian randomization) uses GWAS variants as instrumental variables and gene expression levels or methylation levels as mediating traits, in order to test whether the causal effect of a specific variant on the phenotype-of-interest acts via a specific gene^60^ (but note, in practice, SMR is unable to differentiate between causation and horizontal pleiotropy). SMR incorporates the Heterogeneity in Dependent Instrument (HEIDI) test, which is designed to detect variants with horizontally pleiotropic effects (via their heterogeneity in the SNP-outcome vs. SNP-intermediate trait relationship, in comparison to nearby variants). SMR analyses were repeated after excluding ‘outlier’ variants detected using HEIDI.

#### Test description

The SMR software helps perform two tests. The first is an SMR test, which correlates GWAS effects with eQTL or methylation effects (or any other intermediate trait)^60^. This test suggests causation, although it is unable to fully differentiate between it and pleiotropy. The second test is that of Heterogeneity in Dependent Instrument (HEIDI). This test against the null hypothesis that changes in both eQTL (or other intermediary traits) and the phenotype of interest are caused by one single SNP, which is therefore considered as the candidate for the putative causal effect.

#### Datasets for the SMR analyses: eQTL, cis-mQTL

To perform the above-mentioned tests of causation/pleiotropy, we used three different datasets of association between genetic variants and intermediate traits. The eQTL associations were obtained from the untransformed peripheral blood samples of 5311 subjects^106^ and methylation data from the analyses of LBC methylation of 1980 subjects described elsewhere^107^. They were the largest eQTL datasets available.

### Gene-set enrichment

To identify pathways or other gene sets that were over or under-represented among our results, we used a Gene-Set Enrichment Analysis (GSEA) as implemented in the Meta-Analysis Gene Set Enrichment of Variant (MAGENTA) software^108^. This program assigns scores to each gene based on the strength of association with keratoconus, adjusting for potential confounders such as gene length and linkage disequilibrium. Enrichment for any gene set was assessed within genes above the cut-off of the highest 75th centile of significant gene scores. For the current study, the most recent versions of Gene Ontology (GO), Panther, KGG, Biocarta, and MSigDB databases were used. We also carried out a similar enrichment analysis for the presence of transcription factor binding sites. A permutational procedure and false-discovery rates were used to calculate the significance of enrichment and control for multiple testing.

#### GSEA definitions

For the enrichment analyses, we used updated versions of the GSEA gene sets as described before^109^. We used the versions from November 2018 which were downloaded from http://software.broadinstitute.org/gsea/login.jsp

### Gene expression, GTEx, and other transcription data

We obtained data on tissue expression from several sources for genes located within associated loci. Information about the expression of the genes of interest in systemic (i.e. non-ocular) tissues was obtained from the GTEx Portal for GTEx release v7 (https://gtexportal.org/home/datasets). RNA sequencing data were obtained for both fetal and adult corneal, trabecular meshwork, and ciliary body, as described elsewhere^57^, which we downloaded from the authors’ supplementary information. In addition, we extracted data from the subset of subjects with presumed healthy adult retinas (AMD=1), described elsewhere^110^ that obtained from the GTEx Portal (https://gtexportal.org/home/datasets).

Transcription data were processed using different platforms and were available in different units (Transcripts per Million bases, TPM, for the retina and GTEx tissues, and Fragments per Kilobase, FPKM for the other tissues). For purposes of comparing expression across different tissues for which different methodologies may have been used, expression levels for all tissues were rank-transformed. Hierarchical clustering was used to help visualize similarities and differences of patterns of transcript expression across different tissues (“hclust” package in R).

### LD score regression applied to specifically expressed genes (LDSC-SEG)

Disease-relevant tissues and cell types were identified by analyzing gene expression data together with summary statistics from the meta-analysis of keratoconus in all cohorts, as described elsewhere^59^. Briefly, genes were ranked based on the t-statistic of their expression in each tissue and the 10% most expressed genes for each tissue were considered “specifically expressed genes”. A stratified LD score regression was applied to the meta-analysis summary statistics to evaluate the contribution of the focal genome annotation to trait heritability.

### Prediction analyses

We built a model which included sex, and the major genetic variants associated with keratoconus. The model included all SNPs from a conditional analysis from a European-only conditional analysis^111^, as implemented in the program GCTA^112^ (v1.92.1beta6). The model was trained using the cases and controls of European descent only from the discovery cohort and tested in an independent panel of keratoconus cases and controls. Genotype missingness was < 1% in all cases and controls, and whenever a genotypic value was missing, it was replaced with the population average (calculated on both cases and controls) for each locus. A Receiver Operating Characteristic (ROC) curve was drawn for each case and an Area Under the Curve (AUC) was calculated. R programming language and software environment for statistical computing (https://cran.r-project.org/) was used for both the logistic regression models (‘glm’) and to evaluate the performance of the model (‘ROCR’).

### Reporting summary

Further information on research design is available in the Nature Research Reporting Summary linked to this article.

## Supplementary information

Supplementary Information

Description of Additional Supplementary Files

Supplementary Data 1-14

Supplementary Data 15

Supplementary Data 16

Supplementary Data 17

Supplementary Data 18

Supplementary Data 19

Reporting Summary

## Data Availability

The GWAS summary statistics are available in Supplementary Data 15. The summary statistics are also available through the GWAS Catalogue. Source data for the main figures can be found in Supplementary Data 16 – 19.
