# Supplementary material for: A multi-ethnic genome-wide association study implicates collagen matrix integrity and cell differentiation pathways in keratoconus
Source: Commun Biol. 2021 Mar 1;4:266. doi: 10.1038/s42003-021-01784-0 (PMC7921564; doi:10.1038/s42003-021-01784-0)

A multi-ethnic genome-wide association study implicates collagen matrix integrity and cell differentiation pathways in keratoconus

Alison J Hardcastle, Petra Liskova, Yelena Bykhovskaya, Bennet J McComish, Alice E Davidson, Chris F Inglehearn, Xiaohui Li , Hélène Choquet, Mahmoud Habeeb, Sionne E.M. Lucas, Srujana Sahebjada, Nikolas Pontikos, Karla E. Rojas Lopez, Anthony P. Khawaja, Manir Ali, Lubica Dudakova, Pavlina Skalicka, Bart T.H. Van Dooren, Annette J.M. Geerards, Christoph W. Haudum, Valeria Lo Faro,Abi Tenen, Mark J Simcoe, Karina Patasova, Darioush Yarrand, Jie Yin, Salina Siddiqui, Aine Rice, Layal Abi Farraj, Yii-Der Ida Chen, Jugnoo S. Rah, Ronald M. Krauss, Elisabeth Theusch, Jac C. Charlesworth, Loretta Szczotka-Flynn, Carmel Toomes, Magda A. Meester-Smoor, Andrea J Richardson, Paul A Mitchell, Kent D Taylor, Ronald B. Melles, Anthony J Aldave, Richard A Mills, Ke Cao, Elsie Chan, Mark D Daniell, Jie Jin Wang, Jerome I Rotter, Alex W. Hewitt, Stuart MacGregor, Caroline C.W. Klaver, Wishal D. Ramdas, Jamie E. Craig, Sudha K Iyengar, David O'Brart, Eric Jorgenson, Paul N Baird, Yaron S Rabinowitz, Kathryn P Burdon, Chris J Hammond, Stephen J Tuft, Pirro G Hysi

Contents

Supplementary Figure 1. Plot comparing the effect sizes over keratoconus observed in the discovery and replication stages 2

Supplementary Figure 2. Heat map showing expression of genes located in regions of association 3

Supplementary Figure 3. Violin plots showing expression levels of genes located in regions of association with keratoconus in different eye tissues 4

Supplementary Figure 4. Circular plot overlaying association with keratoconus 5

Supplementary Figure 5. Forest plots showing the effects of the reference alleles 6

Supplementary Figure 6. Projections of the relationship between sample size, number of associated SNPs discovered, and percentage of the variance explained 16

Supplementary Figure 1. Plot comparing the effect sizes over keratoconus observed in the discovery and replication stages. Only loci for which 80% power at the nominal level of significance was considered and therefore represented here. Dots represent loci that were significantly associated with keratoconus at the discovery stage and their colors represent categories of the projected power for association at the replication stage: 0-0.4 (red), 0.4-0.6 (orange), 0-6-0.8 (green) and 0.8-1.0 (blue). The dark line represents a linear regression fit line and the shaded area demarks the 95% Confidence Intervals. The discovery panel (x axis) comprised 2,116 cases and 24,626 controls of European ancestry and the replication panel (y axis) 1,389 cases and 79,727 controls. Polymorphisms identified in the discovery cohort but not shown here were not available for analysis in the replication cohorts.

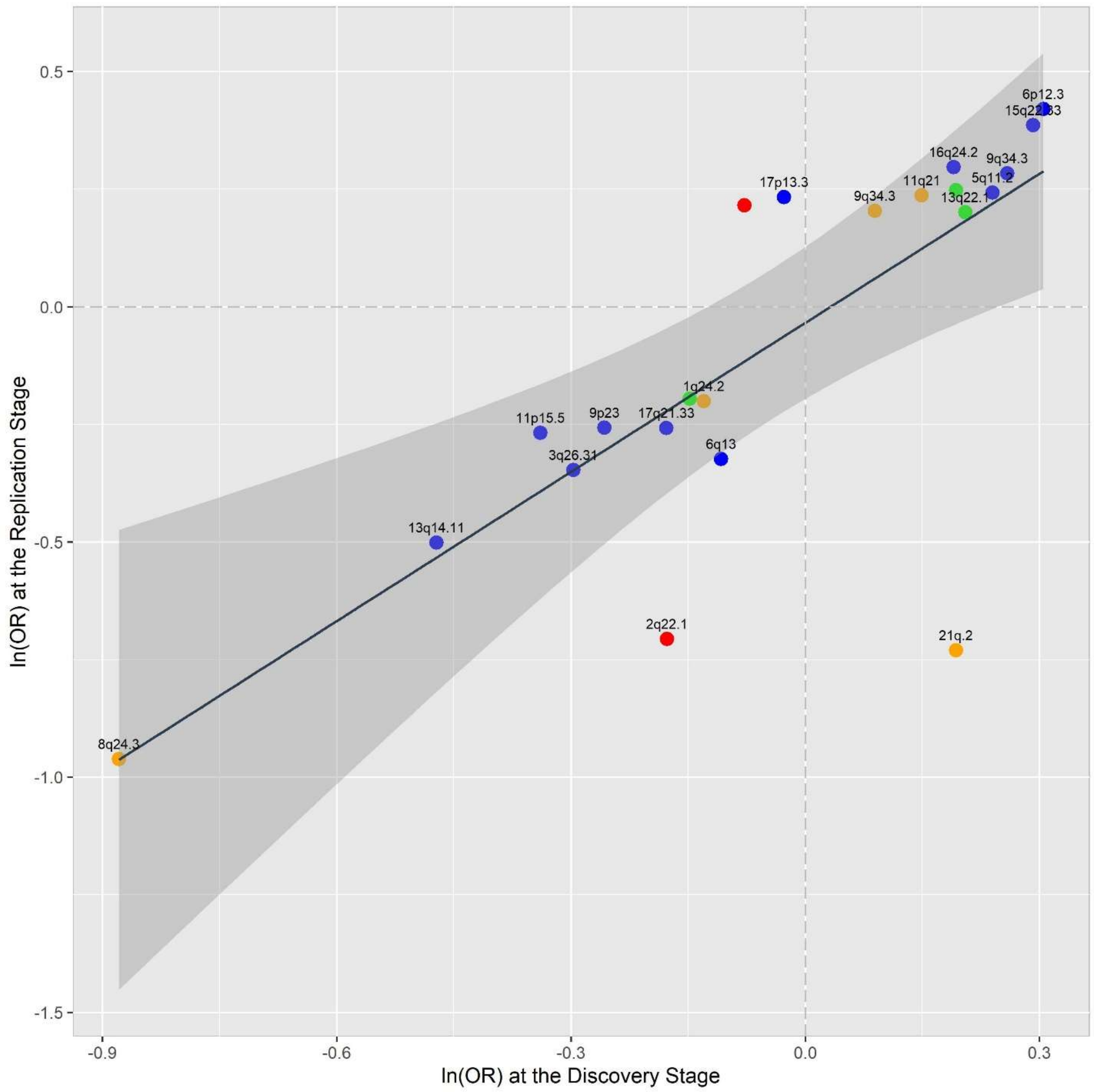

Supplementary Figure 2. Heat map showing expression of genes located in regions of association (x-axis) with keratoconus in different tissues (y-axis). All the genes appearing in Table 1 are included in this figure. The colors represent the centile ranking of the expression level of the gene in the tissue of interest. The hotter colors represent higher ranking of the gene expression and the colder colors low expression. Both genes and tissues are clustered in accordance with their pattern similarity. Fetal eye tissues appear to have similar patterns of gene expressions.

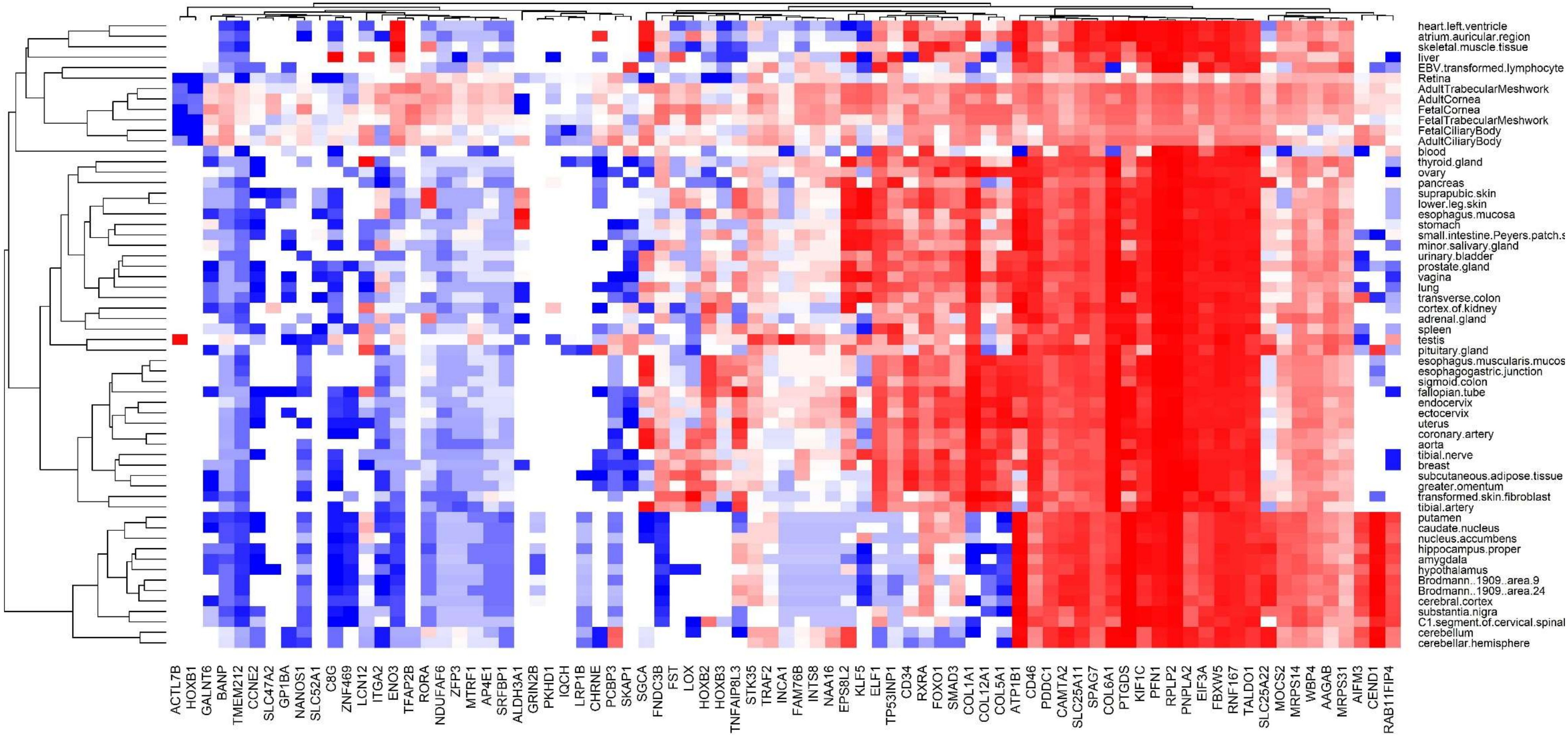

Supplementary Figure 3. Violin plots showing expression levels of genes located in regions of association with keratoconus in different eye tissues. Only genes that are listed in Table 1 and Supplementary Figure 2 were used for the calculations in this figure. The central horizontal bar in the middle of the box within each plot represents the median value, the margins of the white boxes the interquartile range and the margins of the colored violin plots the contours of the distribution in respective tissues. At the bottom of the plot, the value of the median of expression of the different transcripts in the specific tissues is given.

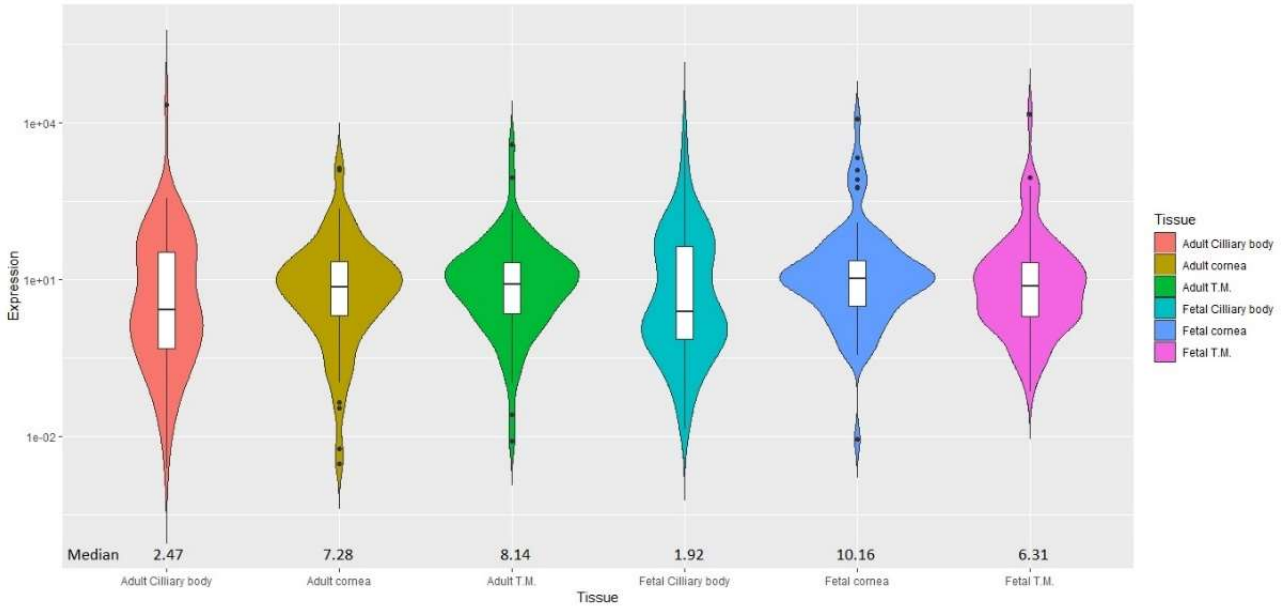

Supplementary Figure 4. Circular plot overlaying association with keratoconus in the meta-analysis (outer circle, (4,669 cases and 116,547), statistically significant ($p<10x^{-06}$) expression Quantitative Trait Locus (eQTL) association with nearest gene (inner layer) and with changes in methylation (computed using data from Westra et al. 2018 in 3,505 cases and 104,353 controls, all of European ancestry) for the same SNPs (middle layer). Each section of the perimeter represents one chromosome and the scale gives the significance level associated with each concentric circle.

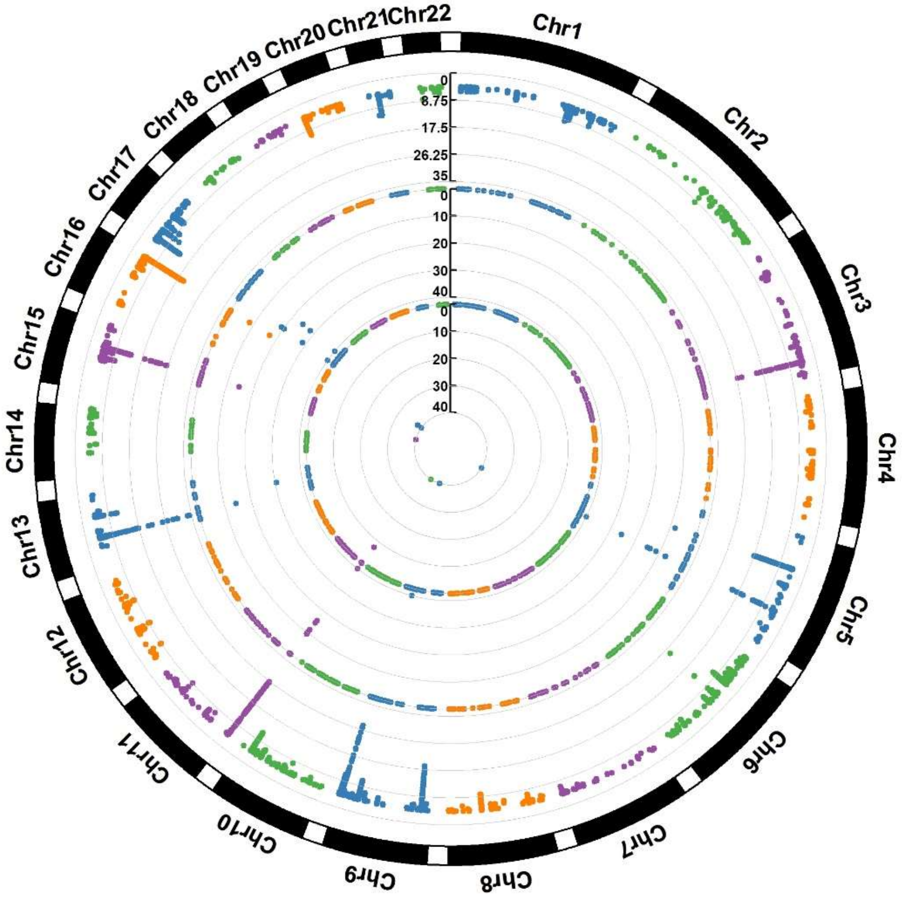

Supplementary Figure 5. Forest plots showing the effects of the reference alleles from the main Table 1 of the manuscript. The SNP and the reference alleles are at he top of each plot; the effects sizes (ln(OR) and the standard errors represented by horizontal bars) in the x-axis and the populations in which they were observed in the y-axis. In some cases the SNPs sucessfully passed quality control analyses in only some of the populations (but always accouning for more than half of the European samples).

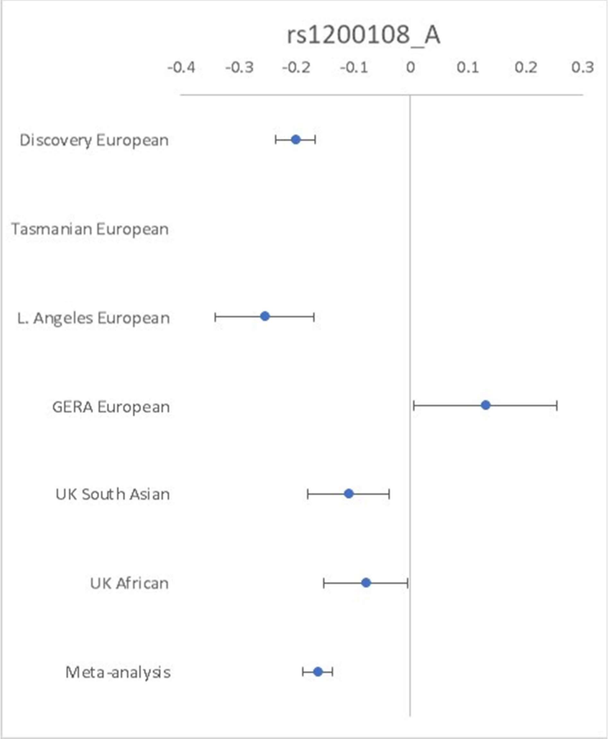

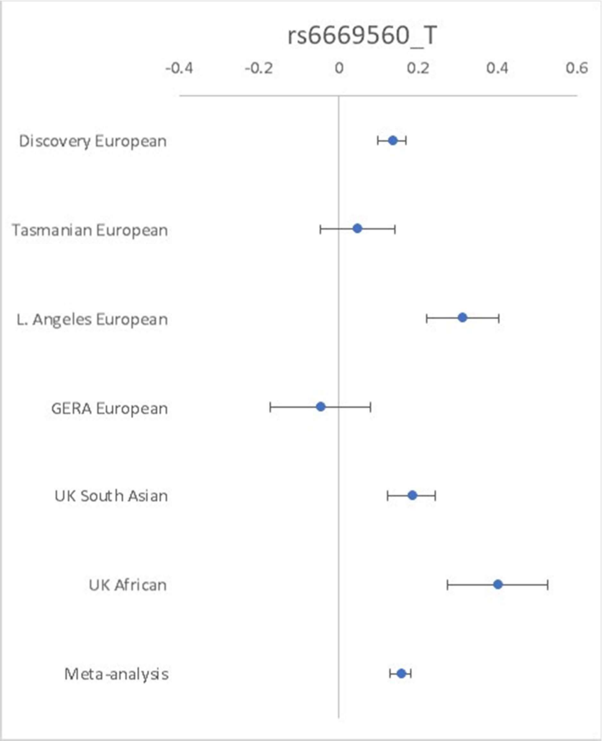

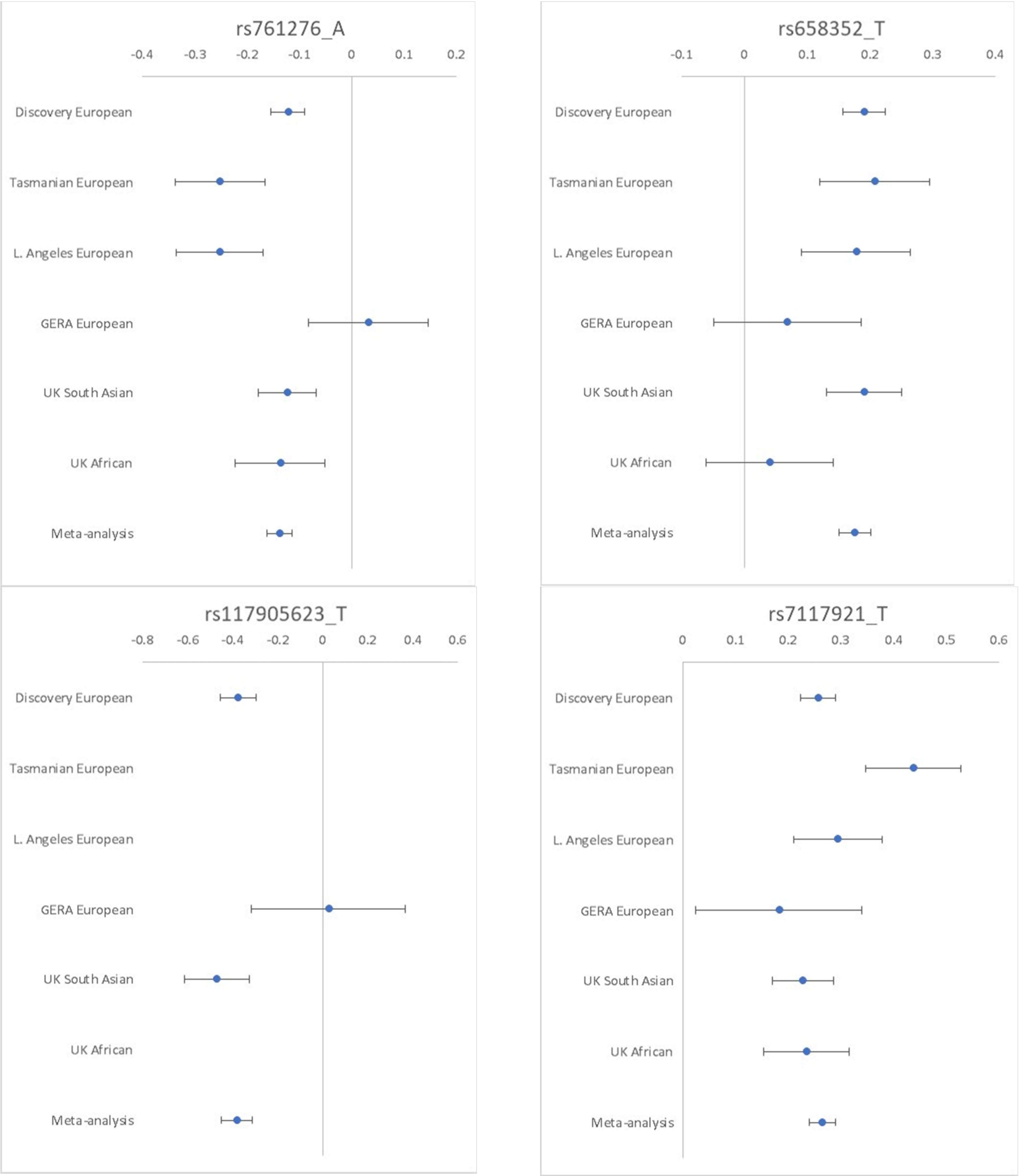
rs761276_A
-0.4 -0.3 -0.2 -0.1 0 0.1 0.2
Discovery European
Tasmanian European
L. Angeles European
GERA European
UK South Asian
UK African
Meta-analysis
rs658352_T
-0.1 0 0.1 0.2 0.3 0.4
Discovery European
Tasmanian European
L. Angeles European
GERA European
UK South Asian
UK African
Meta-analysis
rs117905623_T
-0.8 -0.6 -0.4 -0.2 0 0.2 0.4 0.6
Discovery European
Tasmanian European
L. Angeles European
GERA European
UK South Asian
UK African
Meta-analysis
rs7117921_T
0 0.1 0.2 0.3 0.4 0.5 0.6
Discovery European
Tasmanian European
L. Angeles European
GERA European
UK South Asian
UK African
Meta-analysis

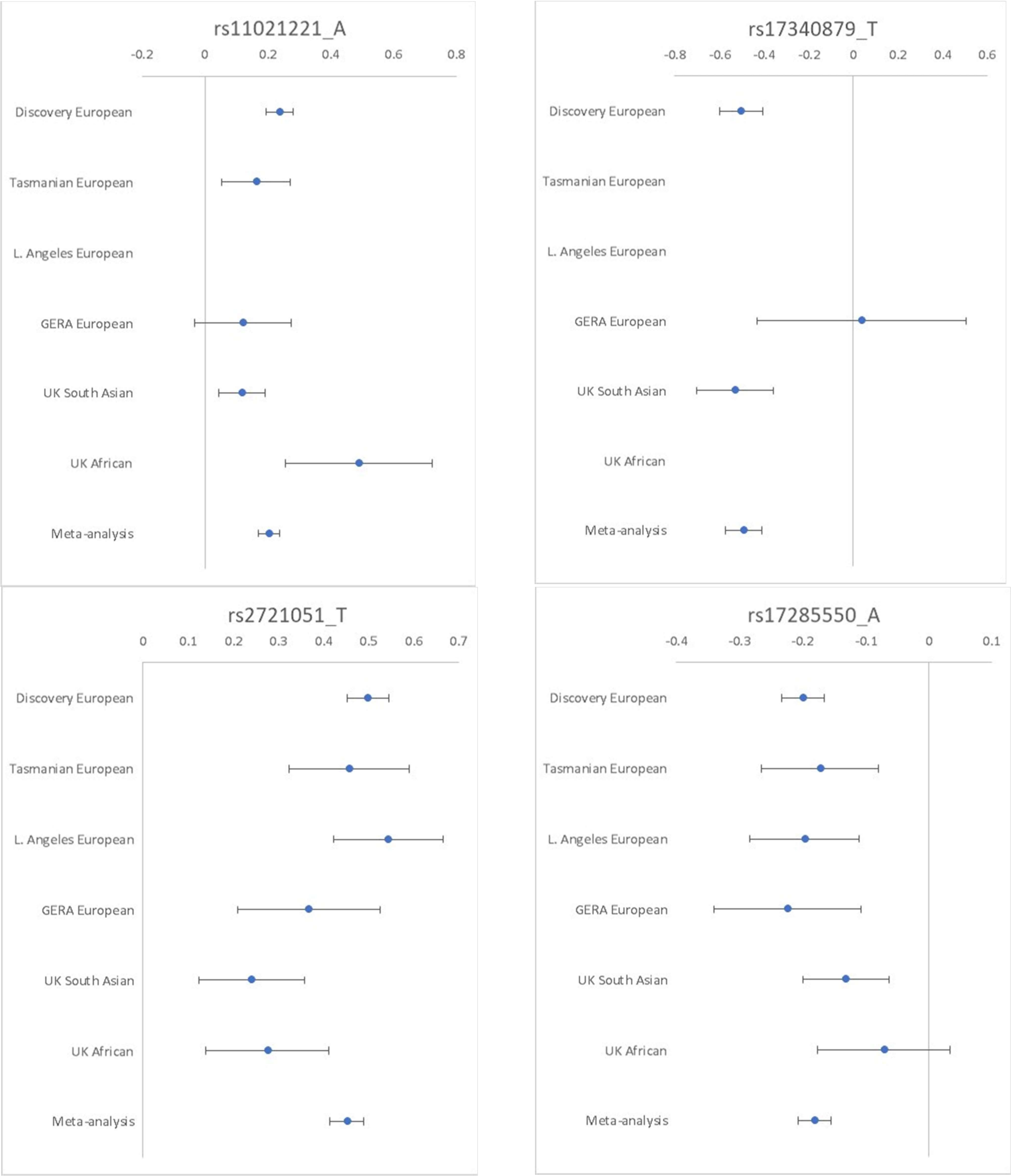
rs11021221_A
-0.2
0
0.2
0.4
0.6
0.8
Discovery European
Tasmanian European
L. Angeles European
GERA European
UK South Asian
UK African
Meta-analysis
rs17340879_T
-0.8
-0.6
-0.4
-0.2
0
0.2
0.4
0.6
Discovery European
Tasmanian European
L. Angeles European
GERA European
UK South Asian
UK African
Meta-analysis
rs2721051_T
0
0.1
0.2
0.3
0.4
0.5
0.6
0.7
Discovery European
Tasmanian European
L. Angeles European
GERA European
UK South Asian
UK African
Meta-analysis
rs17285550_A
-0.4
-0.3
-0.2
-0.1
0
0.1
Discovery European
Tasmanian European
L. Angeles European
GERA European
UK South Asian
UK African
Meta-analysis

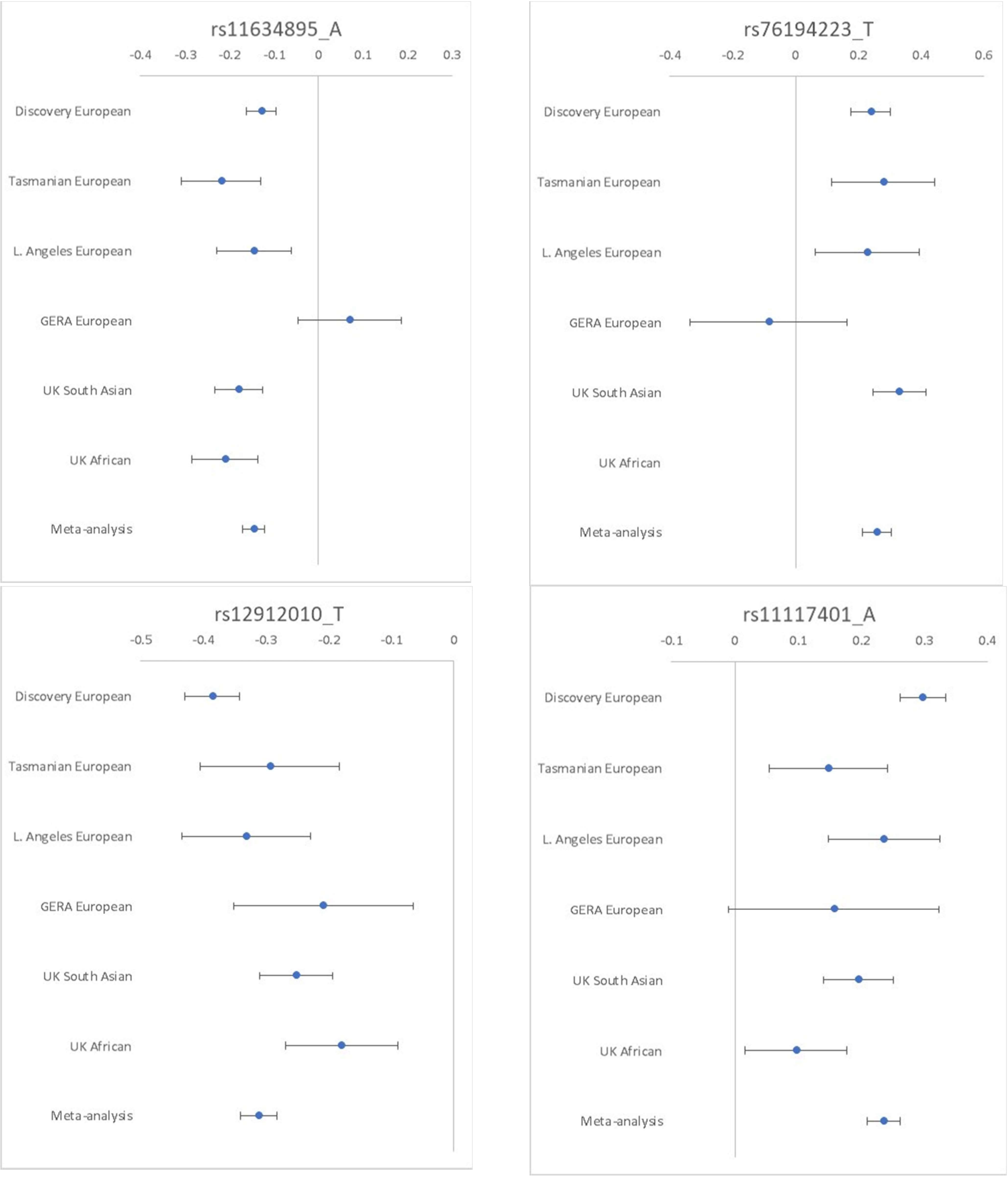
rs11634895_A
-0.4 -0.3 -0.2 -0.1 0 0.1 0.2 0.3
Discovery European
Tasmanian European
L. Angeles European
GERA European
UK South Asian
UK African
Meta-analysis
rs76194223_T
-0.4 -0.2 0 0.2 0.4 0.6
Discovery European
Tasmanian European
L. Angeles European
GERA European
UK South Asian
UK African
Meta-analysis
rs12912010_T
-0.5 -0.4 -0.3 -0.2 -0.1 0
Discovery European
Tasmanian European
L. Angeles European
GERA European
UK South Asian
UK African
Meta-analysis
rs11117401_A
-0.1 0 0.1 0.2 0.3 0.4
Discovery European
Tasmanian European
L. Angeles European
GERA European
UK South Asian
UK African
Meta-analysis

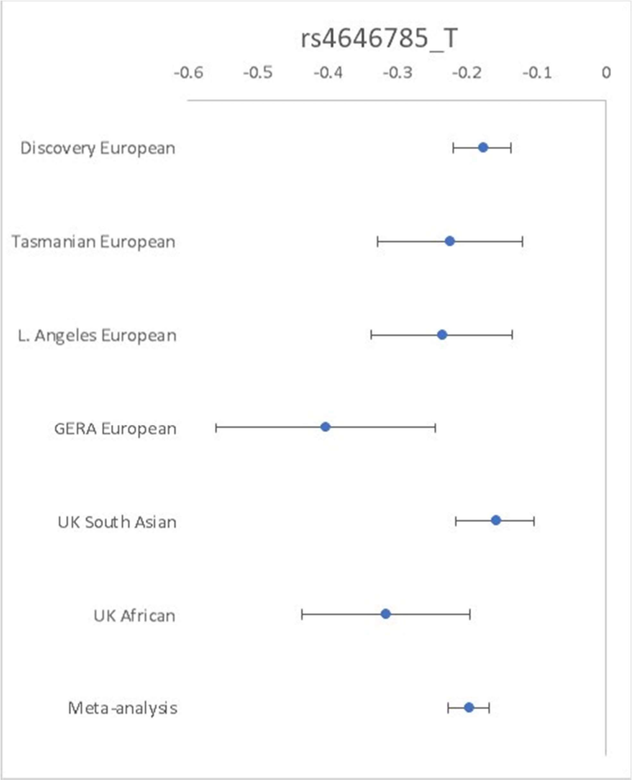
rs4646785_T
-0.6 -0.5 -0.4 -0.3 -0.2 -0.1 0
Discovery European
Tasmanian European
L. Angeles European
GERA European
UK South Asian
UK African
Meta-analysis

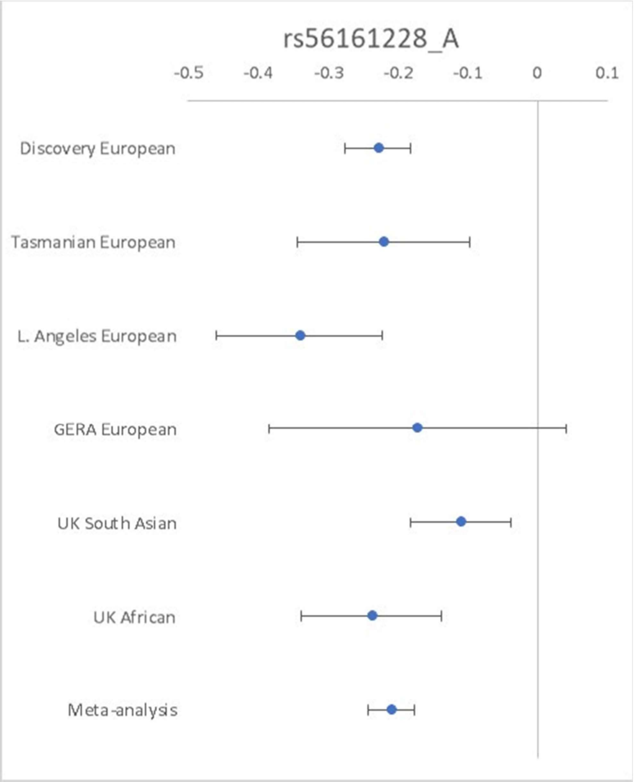
rs56161228_A
-0.5 -0.4 -0.3 -0.2 -0.1 0 0.1
Discovery European
Tasmanian European
L. Angeles European
GERA European
UK South Asian
UK African
Meta-analysis

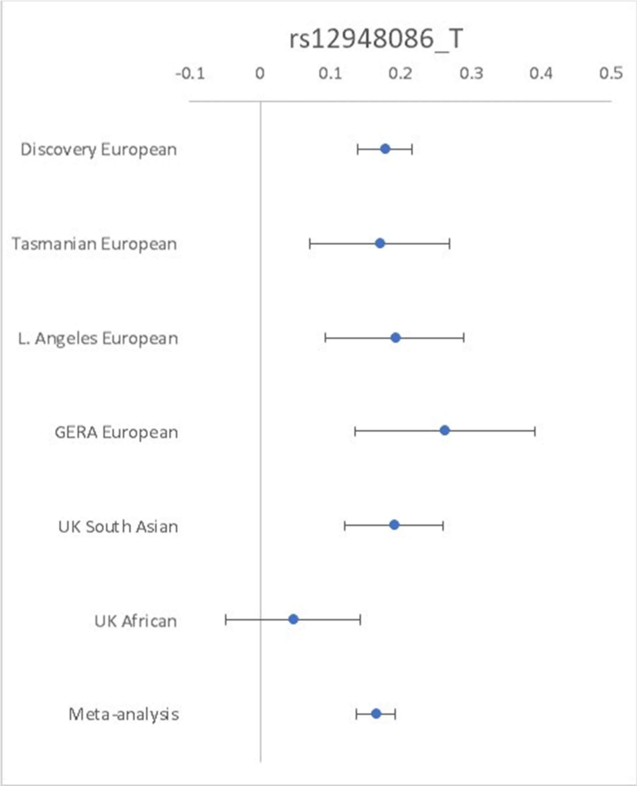
rs12948086_T
-0.1 0 0.1 0.2 0.3 0.4 0.5
Discovery European
Tasmanian European
L. Angeles European
GERA European
UK South Asian
UK African
Meta-analysis

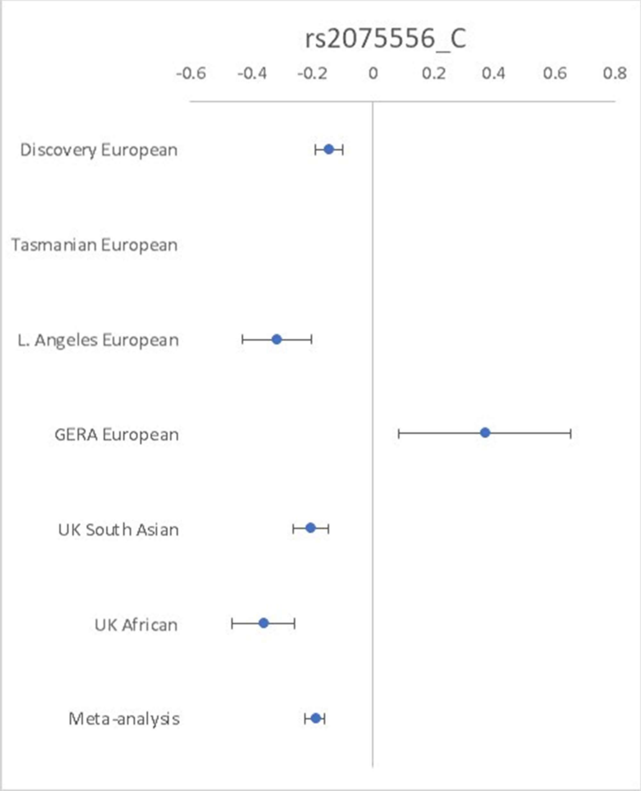
rs2075556_C
-0.6 -0.4 -0.2 0 0.2 0.4 0.6 0.8
Discovery European
Tasmanian European
L. Angeles European
GERA European
UK South Asian
UK African
Meta-analysis

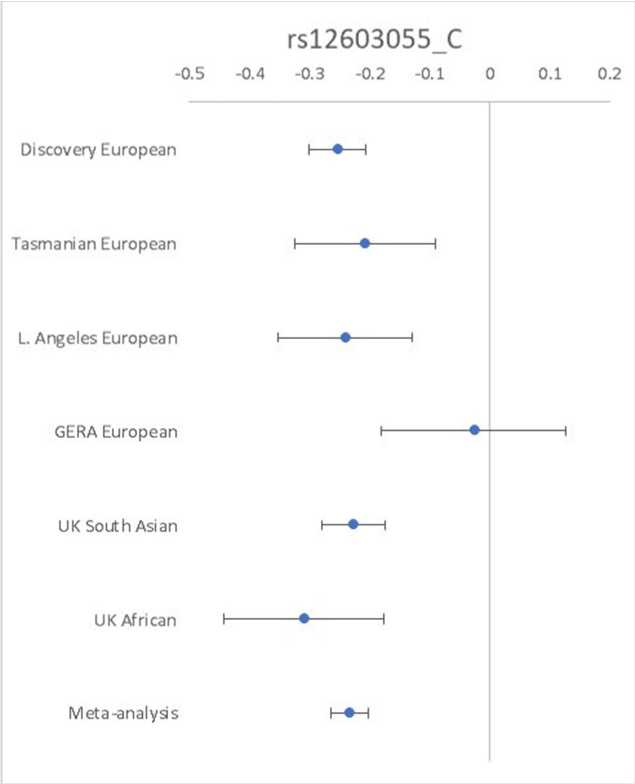
rs12603055_C
-0.5 -0.4 -0.3 -0.2 -0.1 0 0.1 0.2
Discovery European
Tasmanian European
L. Angeles European
GERA European
UK South Asian
UK African
Meta-analysis

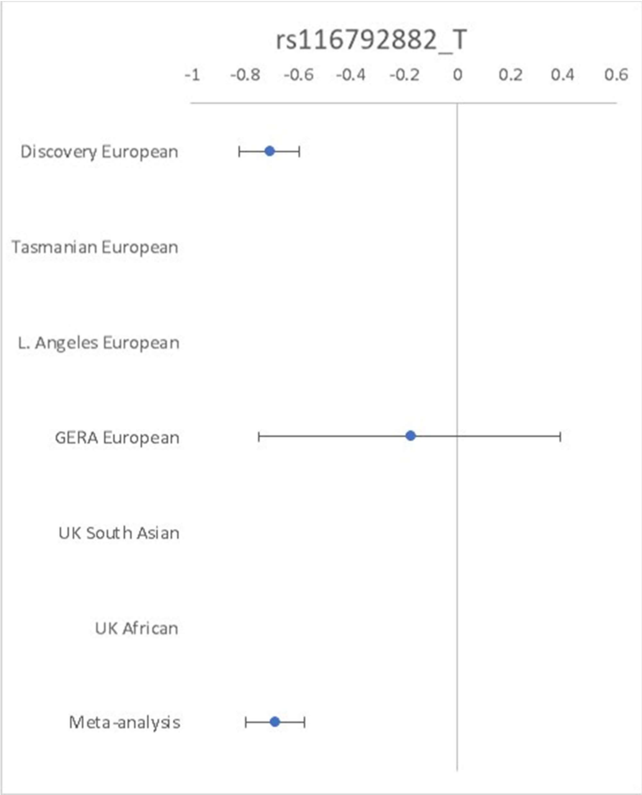
rs116792882_T
-1 -0.8 -0.6 -0.4 -0.2 0 0.2 0.4 0.6
Discovery European
Tasmanian European
L. Angeles European
GERA European
UK South Asian
UK African
Meta-analysis

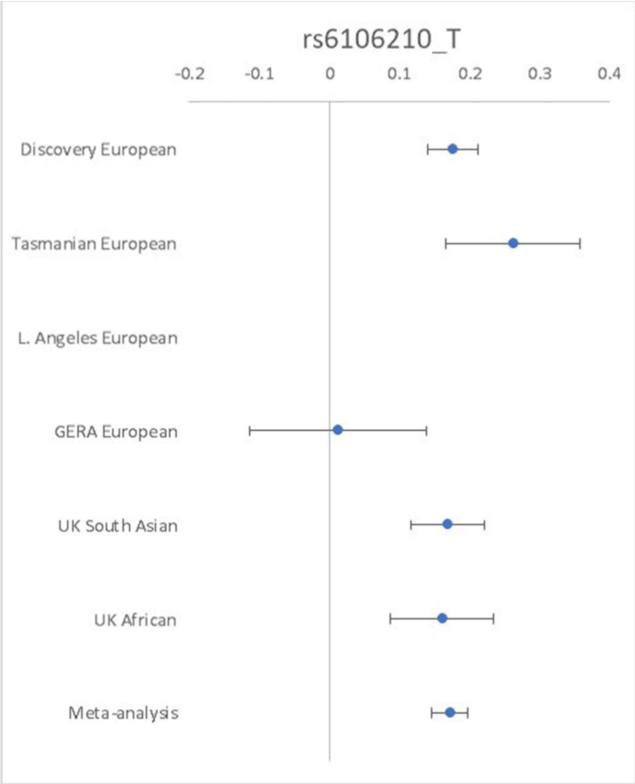
rs6106210_T
-0.2 -0.1 0 0.1 0.2 0.3 0.4
Discovery European
Tasmanian European
L. Angeles European
GERA European
UK South Asian
UK African
Meta-analysis

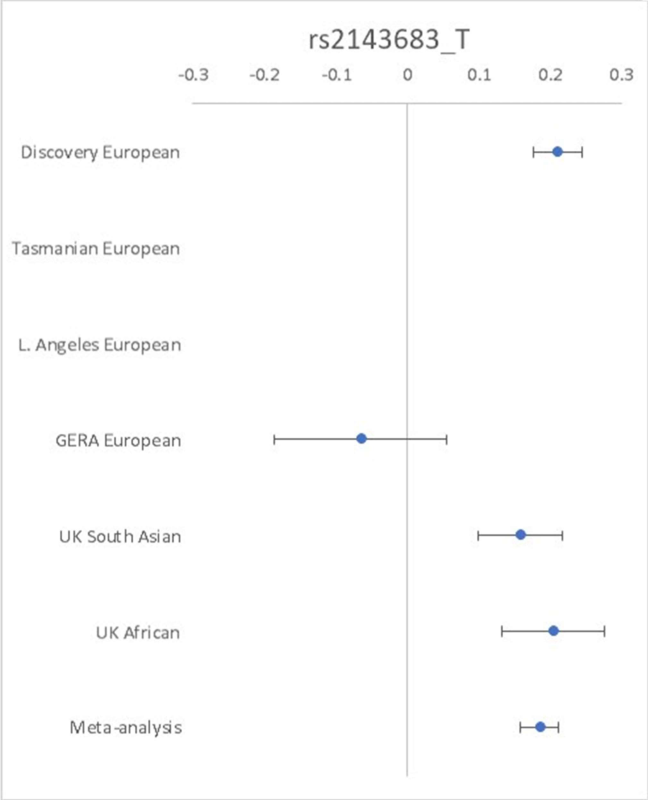
rs2143683_T
-0.3 -0.2 -0.1 0 0.1 0.2 0.3
Discovery European
Tasmanian European
L. Angeles European
GERA European
UK South Asian
UK African
Meta-analysis

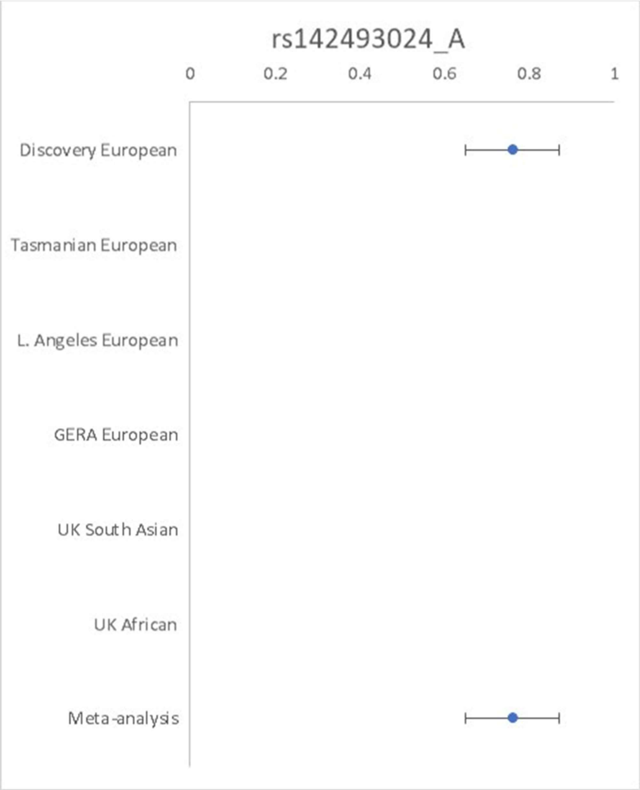
rs142493024_A
0
0.2
0.4
0.6
0.8
1
Discovery European
Tasmanian European
L. Angeles European
GERA European
UK South Asian
UK African
Meta-analysis

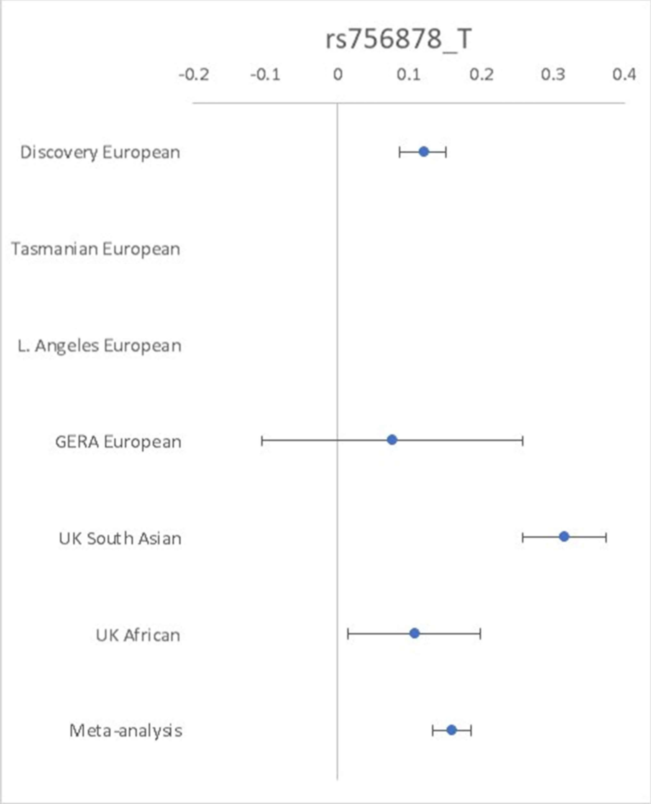
rs756878_T
-0.2
-0.1
0
0.1
0.2
0.3
0.4
Discovery European
Tasmanian European
L. Angeles European
GERA European
UK South Asian
UK African
Meta-analysis

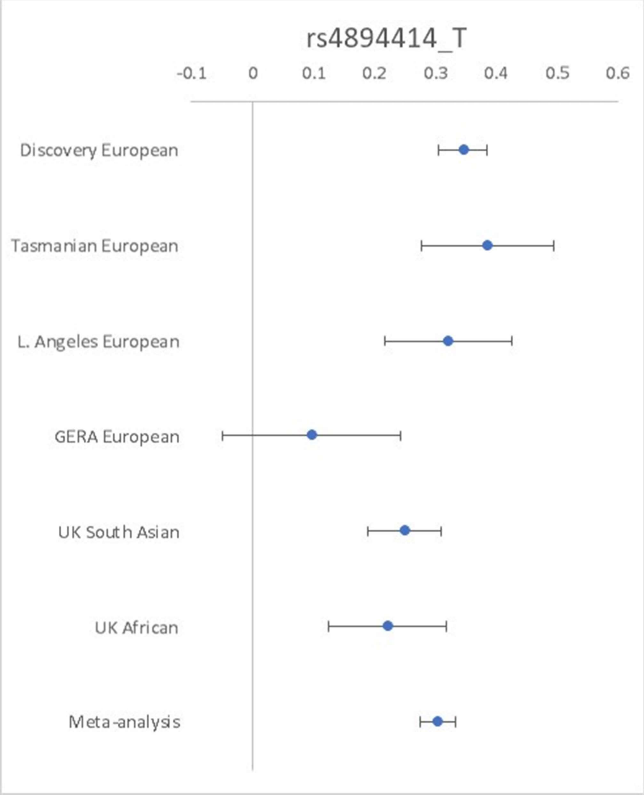
rs4894414_T
-0.1
0
0.1
0.2
0.3
0.4
0.5
0.6
Discovery European
Tasmanian European
L. Angeles European
GERA European
UK South Asian
UK African
Meta-analysis

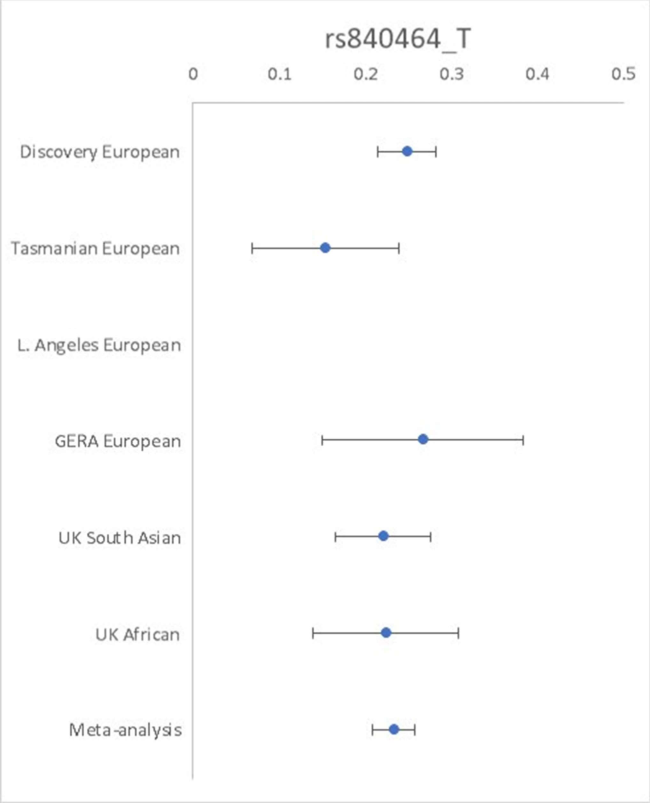
rs840464_T
0
0.1
0.2
0.3
0.4
0.5
Discovery European
Tasmanian European
L. Angeles European
GERA European
UK South Asian
UK African
Meta-analysis

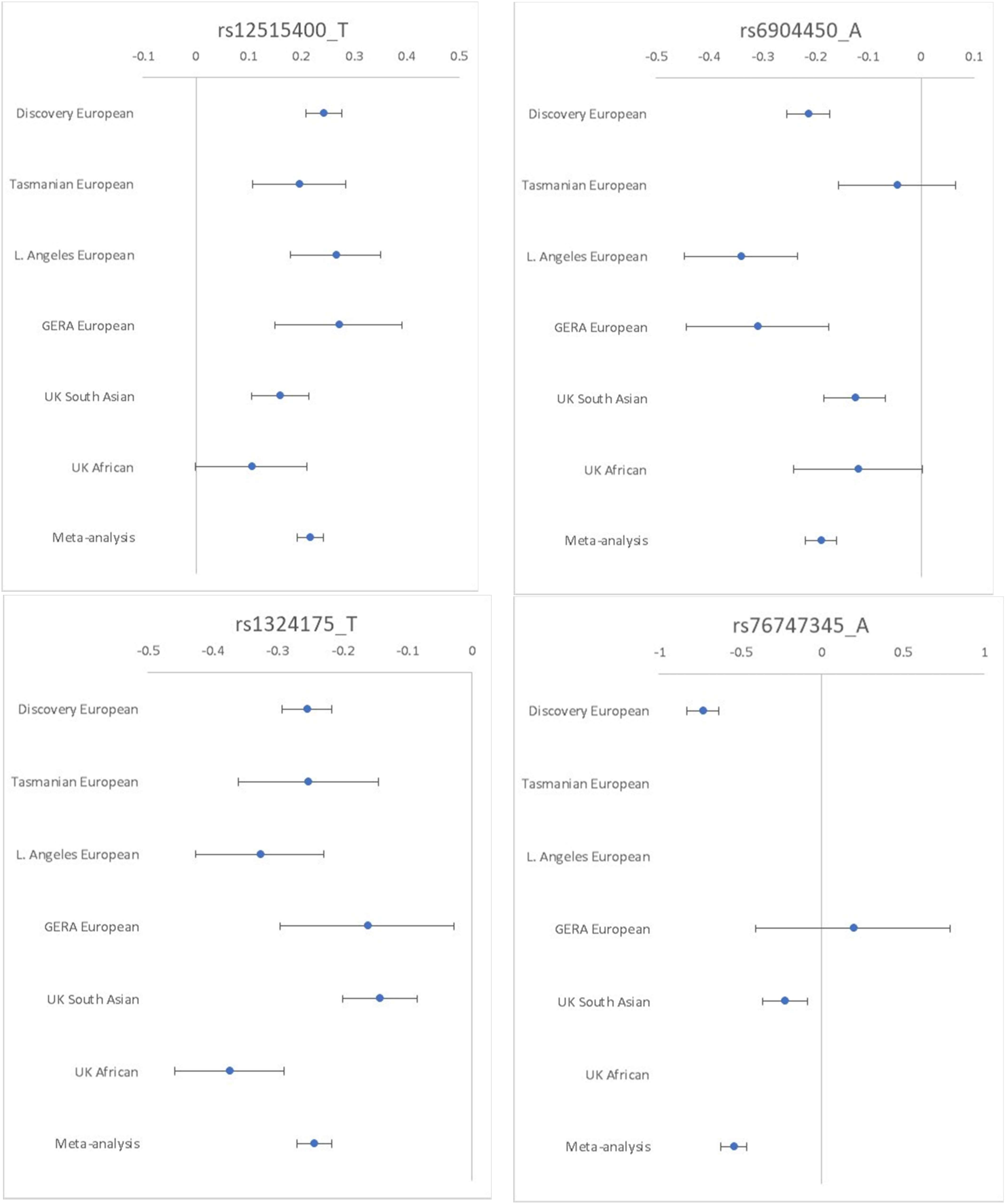
rs12515400_T
-0.1
0
0.1
0.2
0.3
0.4
0.5
Discovery European
Tasmanian European
L. Angeles European
GERA European
UK South Asian
UK African
Meta-analysis
rs6904450_A
-0.5
-0.4
-0.3
-0.2
-0.1
0
0.1
Discovery European
Tasmanian European
L. Angeles European
GERA European
UK South Asian
UK African
Meta-analysis
rs1324175_T
-0.5
-0.4
-0.3
-0.2
-0.1
0
Discovery European
Tasmanian European
L. Angeles European
GERA European
UK South Asian
UK African
Meta-analysis
rs76747345_A
-1
-0.5
0
0.5
1
Discovery European
Tasmanian European
L. Angeles European
GERA European
UK South Asian
UK African
Meta-analysis

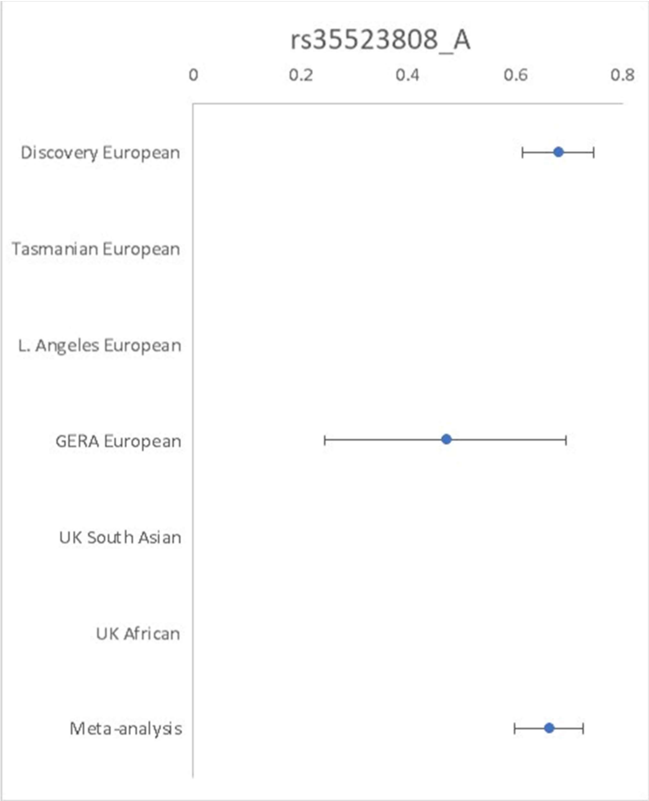
rs35523808_A
0
0.2
0.4
0.6
0.8
Discovery European
Tasmanian European
L. Angeles European
GERA European
UK South Asian
UK African
Meta-analysis

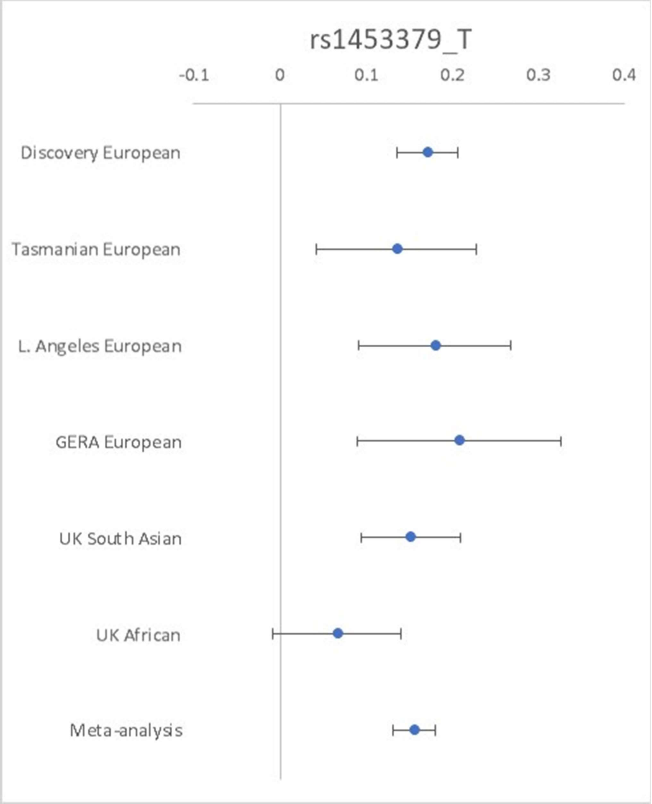
rs1453379_T
-0.1
0
0.1
0.2
0.3
0.4
Discovery European
Tasmanian European
L. Angeles European
GERA European
UK South Asian
UK African
Meta-analysis

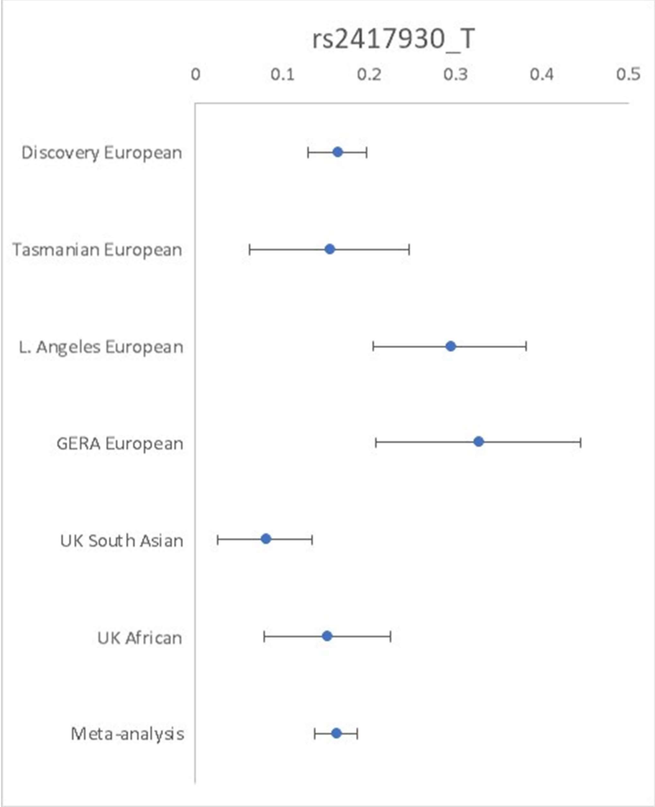
rs2417930_T
0
0.1
0.2
0.3
0.4
0.5
Discovery European
Tasmanian European
L. Angeles European
GERA European
UK South Asian
UK African
Meta-analysis

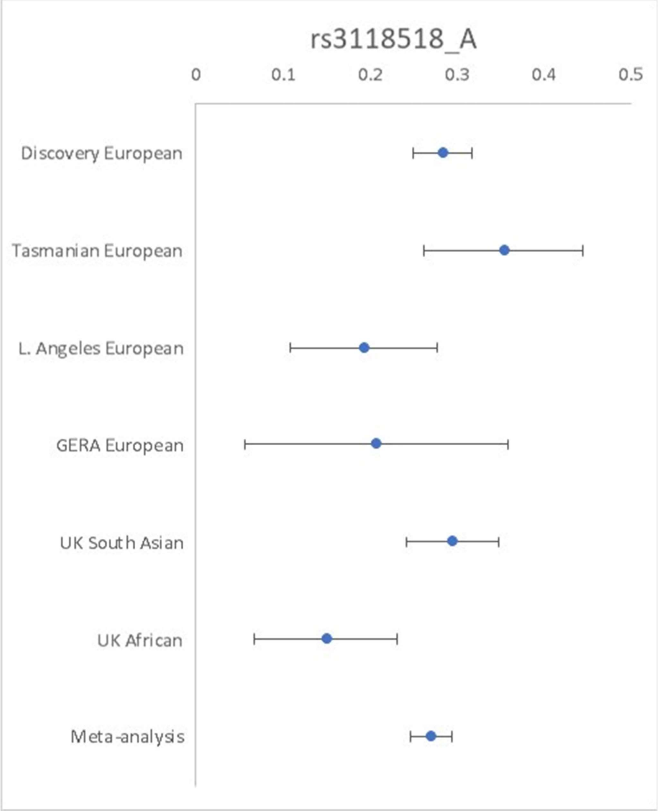
rs3118518_A
0
0.1
0.2
0.3
0.4
0.5
Discovery European
Tasmanian European
L. Angeles European
GERA European
UK South Asian
UK African
Meta-analysis

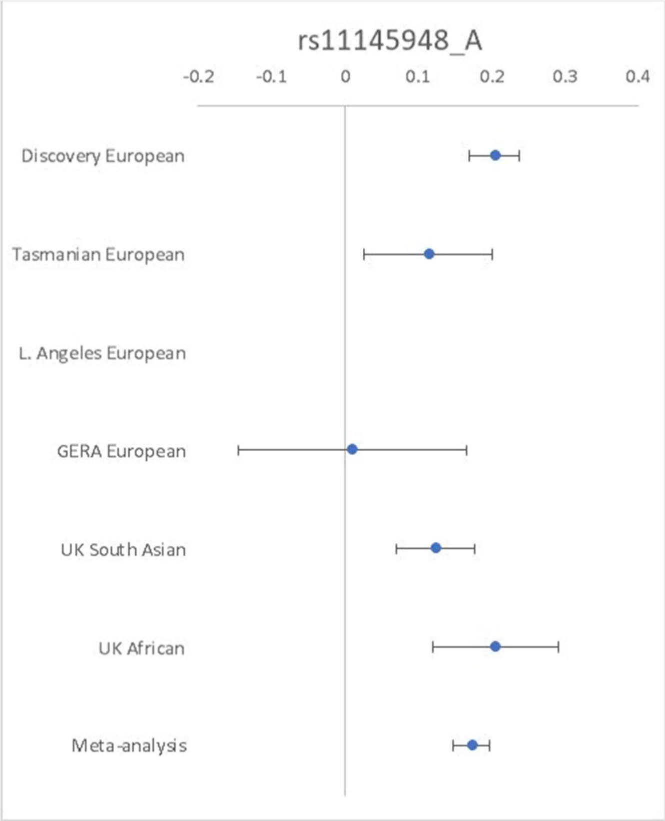
rs11145948_A
-0.2 -0.1 0 0.1 0.2 0.3 0.4
Discovery European
Tasmanian European
L. Angeles European
GERA European
UK South Asian
UK African
Meta-analysis

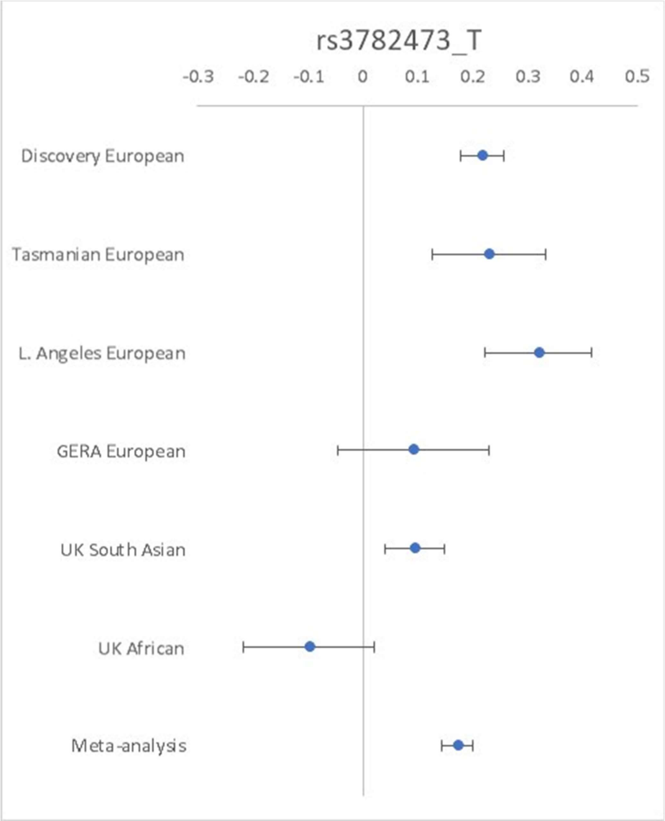
rs3782473_T
-0.3 -0.2 -0.1 0 0.1 0.2 0.3 0.4 0.5
Discovery European
Tasmanian European
L. Angeles European
GERA European
UK South Asian
UK African
Meta-analysis

Supplementary Figure 6. Projections of the relationship between sample size, number of associated SNPs discovered, and percentage of the variance explained. The sample sizes reported here assume a case:control ratio of 10 (i.e. only 10% of the sample sizes in the x-axis would be cases, the rest controls).

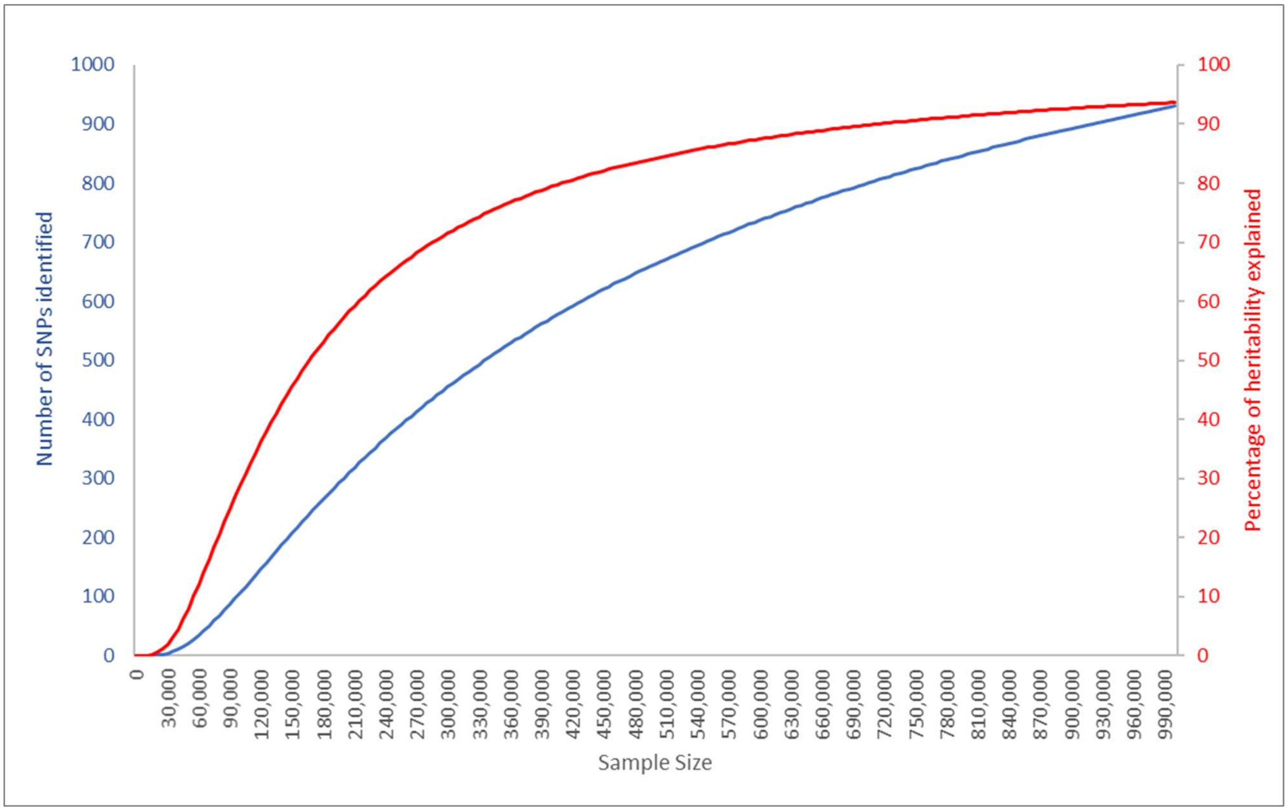

Supplement: Supplementary file 1 — Supplementary Information [file 42003_2021_1784_MOESM1_ESM.pdf]
