# Supplementary material for: A multi-ethnic genome-wide association study implicates collagen matrix integrity and cell differentiation pathways in keratoconus
Source: Commun Biol. 2021 Mar 1;4:266. doi: 10.1038/s42003-021-01784-0 (PMC7921564; doi:10.1038/s42003-021-01784-0)
Supplement: Supplementary file 2 — Description of Additional Supplementary Files [file 42003_2021_1784_MOESM2_ESM.pdf]

Description of Additional Supplementary Files

File Name: Supplementary Data 1 – 14
Description: Supplementary Data tables

Supplementary Data 1: Inflation metrics (lambda) for each participating cohort.

Supplementary Data 2: GWAS results in the European discovery and replication cohorts. The discovery panel comprised 2,116 cases and 24,626 controls of European ancestry and the replication panel 1,389 cases and 79,727 controls.

Supplementary Data 3: Comparison of the results of the discovery cohort and replication cohorts of South Asian and African descents.

Supplementary Data 4: Heterogeneity of genetic association effects across all populations (4,669 cases and 116,547).

Supplementary Data 5: Coding variants that were significantly associated with keratoconus.

Supplementary Data 6: Summary of the most significant eQTL effects.

Supplementary Data 7: Comparison of SNPs previously associated with Fuchs Endothelial Corneal Dystrophy.

Supplementary Data 8: Expression of genes nearest the meta-analysis association signal peak in fetal and adult eye tissues.

Supplementary Data 9: Gene-set enrichment results for Gene Ontology Entries. The GWAS summary statistics were obtained from 4,669 cases and 116,547 controls.

Supplementary Data 10: Results of the LD score regression analysis applied to genes expressed (LDSC-SEG) in multiple tissues from the meta-analysis results.

Supplementary Data 11: SMR tests for mediation of effects through methylation.

Supplementary Data 12: SMR tests for mediation of effects through eQTL effects.

Supplementary Data 13: Genetic correlations between keratoconus and other eye and systemic phenotypes.

Supplementary Data 14: Comparison of the effect sizes from the final keratoconus meta-analysis with CCT effects reported elsewhere.

File Name: Supplementary Data 15
Description: Full Summary Statistics, Used for The Manhattan Plot in The Main Manuscript.

File Name: Supplementary Data 16
Description: Data Used for Figure 1

File Name: Supplementary Data 17
Description: Data Used for Figure 2

File Name: Supplementary Data 18
Description: Data Used for Figure 4

File Name: Supplementary Data 19
Description: Data Used for Figure 5
