# Supplementary material for: A multi-ethnic genome-wide association study implicates collagen matrix integrity and cell differentiation pathways in keratoconus
Source: Commun Biol. 2021 Mar 1;4:266. doi: 10.1038/s42003-021-01784-0 (PMC7921564; doi:10.1038/s42003-021-01784-0)
Supplement: Supplementary file 9 — Reporting Summary [file 42003_2021_1784_MOESM9_ESM.pdf]

Corresponding author(s): Pirro G. Hysi

Last updated by author(s): Sep 9, 2020

Reporting Summary

Nature Research wishes to improve the reproducibility of the work that we publish. This form provides structure for consistency and transparency in reporting. For further information on Nature Research policies, see our Editorial Policies and the Editorial Policy Checklist.

Statistics

For all statistical analyses, confirm that the following items are present in the figure legend, table legend, main text, or Methods section.

n/a	Confirmed	
☐	☒	The exact sample size (*n*) for each experimental group/condition, given as a discrete number and unit of measurement
☒	☐	A statement on whether measurements were taken from distinct samples or whether the same sample was measured repeatedly
☒	☐	The statistical test(s) used AND whether they are one- or two-sided<br>*Only common tests should be described solely by name; describe more complex techniques in the Methods section.*
☐	☒	A description of all covariates tested
☐	☒	A description of any assumptions or corrections, such as tests of normality and adjustment for multiple comparisons
☐	☒	A full description of the statistical parameters including central tendency (e.g. means) or other basic estimates (e.g. regression coefficient) AND variation (e.g. standard deviation) or associated estimates of uncertainty (e.g. confidence intervals)
☒	☐	For null hypothesis testing, the test statistic (e.g. *F*, *t*, *r*) with confidence intervals, effect sizes, degrees of freedom and *P* value noted<br>*Give P values as exact values whenever suitable.*
☒	☐	For Bayesian analysis, information on the choice of priors and Markov chain Monte Carlo settings
☒	☐	For hierarchical and complex designs, identification of the appropriate level for tests and full reporting of outcomes
☒	☐	Estimates of effect sizes (e.g. Cohen's *d*, Pearson's *r*), indicating how they were calculated

Our web collection on statistics for biologists contains articles on many of the points above.

Software and code

Policy information about availability of computer code

Data collection	All cases were recruited in a clinical setting. For the UK based cohorts, controls were selected among participants from the UK Biobank. Other controls for all the populations recruited in the USA, or the replication Australian cohort were recruited among patients that were keratoconus free.
Data analysis	All analyses were conducted using open source software, as specified in the Online Methods.

For manuscripts utilizing custom algorithms or software that are central to the research but not yet described in published literature, software must be made available to editors and reviewers. We strongly encourage code deposition in a community repository (e.g. GitHub). See the Nature Research guidelines for submitting code & software for further information.

Data

Policy information about availability of data

All manuscripts must include a data availability statement. This statement should provide the following information, where applicable:

- Accession codes, unique identifiers, or web links for publicly available datasets
- A list of figures that have associated raw data
- A description of any restrictions on data availability

Summary statistics data will be made available through the GWAS Catalog. They are also available with the paper as Supplementary Data 15.

Field-specific reporting

Please select the one below that is the best fit for your research. If you are not sure, read the appropriate sections before making your selection.

☒ Life sciences ☐ Behavioural & social sciences ☐ Ecological, evolutionary & environmental sciences

For a reference copy of the document with all sections, see nature.com/documents/nr-reporting-summary-flat.pdf

Life sciences study design

All studies must disclose on these points even when the disclosure is negative.

Sample size	This is a meta-analysis of all cases of keratoconus available internationally for research purposes. No preset target, since all samples available were used.
Data exclusions	To minimize confounding effects arising from ethnicity-related heterogeneity, we excluded all samples that were not a full European ancestry, as routinely recommended in similar genetic association studies.<br>In addition, we removed data from participants whose other medical conditions or diagnoses involving the cornea, other than keratoconus.
Replication	Reproducibility of results was assured by comparing association results, effect size and direction as well as significance of association tests among several population-based cohorts.
Randomization	No randomization procedure was needed.
Blinding	No blinding was needed.

Reporting for specific materials, systems and methods

We require information from authors about some types of materials, experimental systems and methods used in many studies. Here, indicate whether each material, system or method listed is relevant to your study. If you are not sure if a list item applies to your research, read the appropriate section before selecting a response.

Materials & experimental systems

n/a	Involved in the study	
☒	☐	Antibodies
☒	☐	Eukaryotic cell lines
☒	☐	Palaeontology and archaeology
☒	☐	Animals and other organisms
☐	☒	Human research participants
☐	☒	Clinical data
☒	☐	Dual use research of concern

Methods

n/a	Involved in the study	
☒	☐	ChIP-seq
☒	☐	Flow cytometry
☒	☐	MRI-based neuroimaging

Human research participants

Policy information about studies involving human research participants

Population characteristics	Regression-based association analyses had refractive error as the outcome, number of alleles at each polymorphic locus as predictors, making adjustment for sex and the most important principal components.
Recruitment	All cases were recruited in a clinical setting. For the UK based cohorts, controls were selected among participants from the UK Biobank. Other controls for all the populations recruited in the USA, or the replication Australian cohort were recruited among patients that were keratoconus free.
Ethics oversight	The Moorfields Eye Hospital Research Ethics Committee (09/H0721/19); Leeds East Research Ethics Committee (reference 10/H1306/63); the Royal Victorian Eye and Ear Hospital Human Research and Ethics Committee (Project#10/954H); Southern Adelaide Clinical Human Research Ethics Committee (HREC), the HREC of the Royal Victorian Eye and Ear Hospital and the Health and Medical HREC of the University of Tasmania; nstitutional Review Board of the Kaiser Foundation Research Institute for the GERA study.

Note that full information on the approval of the study protocol must also be provided in the manuscript.

Clinical data

Policy information about clinical studies

All manuscripts should comply with the ICMJE guidelines for publication of clinical research and a completed CONSORT checklist must be included with all submissions.

Clinical trial registration	Not applicable (not a clinical study)
Study protocol	N/A
Data collection	N/A
Outcomes	N/A
